# Impairment of APPL1/Myoferlin facilitates adipogenic differentiation of mesenchymal stem cells by blocking autophagy flux in osteoporosis

**DOI:** 10.1007/s00018-022-04511-y

**Published:** 2022-08-19

**Authors:** Yunhui Zhang, Wenjie Liu, Weiquan Yuan, Zhaopeng Cai, Guiwen Ye, Guan Zheng, Chenhao Xu, Xinglang Wang, Chenying zeng, Rujia Mi, Pei Feng, Fenglei Chen, Yanfeng Wu, Huiyong Shen, Peng Wang

**Affiliations:** 1 Department of Orthopedics, The Eighth Affiliated Hospital, Sun Yat-Sen University, 3025# Shennan Road, Shenzhen, 518000 People’s Republic of China; 2 Center for Biotherapy, The Eighth Affiliated Hospital, Sun Yat-Sen University, 3025# Shennan Road, Shenzhen, 518000 People’s Republic of China

**Keywords:** APPL1, Mesenchymal stem cells, Adipogenic differentiation, Osteoporosis, Myoferlin, Autophagy

## Abstract

An imbalance of human mesenchymal stem cells (hMSCs) adipogenic and osteogenic differentiation is crucial in the pathogenesis of osteoporosis, and elucidation of the underlying mechanism is urgently needed. APPL1, an adaptor protein of the adiponectin receptor, was recently shown to be closely related to bone mass. However, the role of APPL1 in the imbalance of hMSC differentiation in osteoporosis is unclear. Therefore, we aimed to explore the mechanisms by which APPL1 alters hMSCs adipogenic differentiation in osteoporosis. Here, we found that APPL1 expression was downregulated in elderly patients with osteoporosis and in mouse osteoporosis model. APPL1 negatively regulated hMSC adipogenic differentiation in vivo and in vitro. Mechanistically, by enhancing ubiquitination-mediated Myoferlin degradation, downregulated APPL1 expression increased the risk of lysosome dysfunction during hMSCs adipogenic differentiation. Lysosomal dysfunction inhibited autophagy flux by suppressing autophagosome degradation and promoted hMSC differentiation towards the adipocyte lineage. Our findings suggest that APPL1/Myoferlin downregulation promoted hMSCs adipogenic differentiation by inhibiting autophagy flux, further impairing the balance of hMSCs adipogenic and osteogenic differentiation in osteoporosis; the APPL1/ Myoferlin axis may be a promising diagnostic and therapeutic target for osteoporosis.

**Supplementary Information:**

The online version contains supplementary material available at 10.1007/s00018-022-04511-y.

## Introduction

Osteoporosis is a skeletal disease characterized by a low bone mass and increasing bone fragility. With the progression of osteoporosis, brittle fractures become more common, resulting in a substantial threat to the patient’s quality of life [1]. Recent studies have reported a negative relationship between bone marrow adipose tissue and bone mineral density (BMD). An increased bone marrow adipose tissue content may be related to a lower bone mass. People with osteoporosis have a higher fat content and greater number of fat cells in their bone marrow than healthy people [2, 3]. This disorder is caused by the abnormal differentiation of adipocytes and osteoblast progenitor cells.

Human bone marrow mesenchymal stem cells (hMSCs) are pluripotent stem cells that show self-renewal and multidirectional differentiation [4]. The balance between adipogenic and osteogenic differentiation of hMSCs, which are common progenitor cells of osteoblasts and bone marrow adipocytes, in time and space is the key to maintaining bone health, and differentiation towards one phenotype will inhibit differentiation towards the other phenotypes [5–7]. Recent studies have shown an imbalance between hMSCs adipogenic and osteogenic differentiation in patients with osteoporosis, and hMSCs in bone marrow tend to differentiate into adipocytes [2, 8]. The number of adipocytes in the bone marrow increases abnormally, while the number of osteoblasts decreases, resulting in bone mass loss [9]. However, at present, the underlying mechanisms by which the differentiation of hMSCs shifts towards the adipocyte lineage in patients with osteoporosis are unclear.

Adaptor protein containing pH domain, PTB domain, and leucine zipper motif 1 (APPL1) is an adaptor protein of the adiponectin receptor (ApnR) with three different domains, and APPL1 directly binds to ApnRs following adiponectin (APN) stimulation [10]. APPL1 is a functional protein that plays an important role in APN signal transduction, as well as a key regulator of the interaction between the APN pathway and insulin pathway [11]. As one of the most abundant adipose hormones, APN plays an important role in regulating energy metabolism and insulin sensitivity [12, 13]. Previously, APN was presumed to be solely produced and released in adipose tissue, but recent research has revealed that APN and its receptor are expressed in osteocytes, osteoblasts, and their progenitors [14]. In addition, numerous studies have shown a correlation between APN levels and bone mass, indicating that APN is involved in the metabolism and evolution of bone and its associated cells. However, MSCs are a common source of adipocytes and osteoblasts in bone tissue, and the molecular mechanism by which APN and its receptor regulate differentiation remains unclear. Changes in APN levels and their related pathways may affect the bone content by influencing the differentiation trajectory of hMSCs [15].

Myoferlin (MYOF), a protein identified in muscle cells, is a member of the Ferlin family that is involved in the membrane vesicle transport system in cells, including vesicle transport, membrane fusion, and membrane repair [16–18]. Recently, the function of MYOF through membrane repair that stabilizes organelles has attracted increasing attention. Previous studies have shown that MYOF is expressed at high levels in tumour cells and prevents lysosomal damage in tumour cells, thereby prolonging their lifespan [19]. Lysosomes are vital organelles in cells, and inactivated macromolecules, degraded organelles and extracellular substances are transported into the lumen of lysosomes via endocytosis and autophagy, where they are digested with numerous acid hydrolases [20, 21]. An intact lysosome is closely related to the process of autophagy. Autophagy is a lysosomal degradation pathway that is essential for cellular homeostasis, survival and differentiation. Autophagy, which serves as the "scavenger" of cells, performs various critical functions, such as replacing intracellular proteins and organelles, regulating metabolic activities and maintaining cellular homeostasis [22, 23]. The autophagy process depends on the formation, maturation and relocation of autophagosomes, which finally fuse with lysosomes. Lysosome dysfunction affects the autophagic degradation mechanism, and the level of autophagy is correspondingly altered [24]. Recent studies have shown that autophagy plays a key role in regulating bone metabolism, and the level of autophagy modifies the trend of the lineage differentiation of hMSCs [25]. Furthermore, the stability of lysosome function is critical for sustaining the functions of the mTORC1 and AMPK pathways, and alterations in these key pathways will result in abnormal physiological functions of hMSCs [26, 27].

In this study, we aimed to explore the mechanism underlying the role of APPL1 in the abnormal balance of hMSC adipogenic and osteogenic differentiation in osteoporosis. MYOF was downstream of APPL1, affecting the level of autophagy by modulating lysosomal function and then suppressing the adipogenic differentiation of hMSCs. Thus, the APPL1/MYOF axis may be a promising target for the clinical diagnosis and treatment of osteoporosis.

## Materials and methods

### MSC isolation and culture

Eighteen healthy donors aged between 20 and 30 years were recruited after they were informed of the potential risks and after obtaining informed consent and consent to publish the data. The study was approved by the Ethics Committee of The Eighth Affiliated Hospital, Sun Yat-sen University (Clinical ethical approval No. 2021r209). Using our previously reported methods, hMSCs were isolated and purified from the bone marrow, which was extracted from the posterior superior iliac spine under sterile conditions. hMSCs were cultured at 37 °C with Dulbecco's modified Eagle's medium (Gibco, USA) and 10% foetal bovine serum (Gibco, USA). The culture medium was replaced every 2–3 days. Upon reaching 80–90% confluence, hMSCs were passaged into two flasks or seeded into a 12-well plate for subsequent experiments by trypsin digestion.

Murine bone marrow mesenchymal stem cells were obtained from mice with osteoporosis (16-month-old mice or mice that underwent ovariectomy) and control (8-month-old mice or mice that underwent sham surgery) C57BL/6J mice. Briefly, the femora and tibiae were isolated, and the tissue surrounding the bone was cleaned. Bone marrow was flushed from the medullary cavity with a syringe into complete α-MEM (Gibco) containing 10% foetal bovine serum and 100 IU/ml penicillin–streptomycin. Afterwards, the cell suspension was collected and plated in a cell culture flask. The medium was changed every 3 days, and MSCs were passaged into two flasks or seeded in a 12-well plate for subsequent experiments by trypsin digestion.

### Collection of human bone samples

Osteoporosis samples were acquired from patients with postmenopausal osteoporosis who underwent surgery, and normal control samples were acquired from patients who underwent surgery after an accident. The criteria for nonosteoporosis included nonpostmenopausal women aged ≤ 50 years with BMD *T*-score ≥ − 1. The criteria for osteoporosis included postmenopausal women aged > 50 with BMD *T*-score ≤  − 2.5 or who underwent fragility fracture with BMD *T*-score > − 2.5 and < − 1 [28, 29]. After obtaining informed consent and consent to publish the data, we acquired the femoral heads from patients and used them in our research. The detailed characteristics of the patients included in our study are shown in the supplementary materials.

### Western blotting

Cells were lysed in RIPA buffer containing a 1% phosphatase and protease inhibitor cocktail for 30 min on ice. Cell lysates were collected and then centrifuged 14,000 rpm for 30 min at 4 °C. After collecting the protein supernatant, the protein concentration was measured and quantified using a BCA protein assay kit. Equal amounts of protein were mixed with SDS–PAGE loading buffer, separated by SDS polyacrylamide gel electrophoresis and transferred to polyvinylidene fluoride membranes. Five percent nonfat milk was used to block the membranes for 1 h. Then, the membranes were incubated overnight at 4 °C with primary antibodies against APPL (1:2000, Abcam Cat# ab180140), MYOF (1:2000, Abcam Cat# ab178386), PPAR-γ (1:1000, Abcam Cat# ab178860), C/EBPα (1:1000, Abcam Cat# ab40764), FABP4 (1:1000, Abcam Cat# ab92501), Ubiquitin (1:2000, Abcam Cat# ab134953), LAMP1 (1:1000, Abcam Cat# ab278043), DYKDDDDK Tag (1:1000, Cell Signaling Technology Cat# 8146) and GAPDH (1:1000, Cell Signaling Technology Cat# 5174). Afterwards, the membranes were washed with Tris-buffered saline-Tween (TBST) 3 times to eliminate nonspecific binding and incubated with HRP-conjugated secondary antibodies at room temperature for 1 h. After 3 washes, chemiluminescent HRP substrate was applied to detect the target protein bands, and the densitometry analysis was performed with ImageJ software.

### Real-time quantitative reverse transcription-polymerase chain reaction (qRT–PCR)

After 3 washes with phosphate-buffered saline (PBS), TRIzol (Thermo) was used to extract total RNA from the cells. RNA was reverse transcribed into complementary DNA using a PrimeScript RT Reagent Kit (TaKaRa). qRT–PCR was performed using SYBR Green Premix Ex Taq (TaKaRa) according to the manufacturer's protocol. A real-time PCR system was used to detect the gene expression levels, and the relative expression was analysed and calculated using the 2^−ΔΔCt^ method, with GAPDH serving as the normalization control. Primers were designed. The primers used for detecting mRNA expression in our study are shown in Supplementary Table S1.

### RNA interference

Small interfering RNAs (siRNAs) targeting APPL1 and MYOF and a negative control were purchased from IGEbio (Guangzhou, China). When the cells reached 60–80% confluence, hMSCs were transfected with Opti-MEM reduced serum medium, Lipofectamine™ RNAiMAX (Invitrogen) and siRNA cocktail (1 OD per 1.8 × 10^6^ cells) according to the manufacturer’s instructions. At 5 h post-transfection, the transfection medium was removed. The detection of the knockdown efficiency and subsequent experiments were performed after 48 h, and siRNA was used to knock target genes again on day 6 of adipogenic or osteogenic induction. The sequences of the siRNAs used in this study are listed in (Supplementary Tables S2–S4).

### Lentivirus, adenovirus, plasmid construction and infection

All plasmids, including pcDNA3.1( +)-Flag-APPL1, pcDNA3.4( +)-Flag-BAR, pcDNA3.4( +)-Flag-PH and pcDNA3.4( +)-Flag-PTB were constructed by IGEbio. According to the manufacturer’s instructions Lipofectamine 3000 (Invitrogen) was used to transfect 293 T cells seeded into 6-well plates at 4 × 10^5^ cells/well. Briefly, 5 µl of P3000 and 5 µl of Lipo3000 were mixed with cDNA, and finally added to the plates.

All lentiviruses, including the vector control, mCherry-EGFP-LC3B fusion protein lentivirus, APPL1 overexpression adenovirus and APPL1 overexpression lentivirus, were designed and synthesized by OBiO Technology (Shanghai). hMSCs were incubated with premixed medium containing lentivirus (MOI = 30), 5 μg/ml polybrene and complete medium for 24 h. The detection of transfection efficiency and subsequent experiments were performed after 48 h. After the osteoporosis mouse model was constructed on day 7, adenoviruses were used to infect the mice.

### Adipogenic differentiation assay in vivo

This experiment was performed as described in a previous report [30]. Briefly, hMSCs were incubated with adipogenic differentiation medium for 5 days after transfection with the lentivirus and siRNA in vitro. Cells were collected and mixed with 200 μl of Matrigel (BD Biosciences, USA); then, the mixture was injected subcutaneously into the backs of 8-week-old BALB/c-nu/nu male nude mice (Gempharmatech, China). After 6–8 weeks, the cells/Matrigel implants were harvested and fixed with 4% paraformaldehyde for 24 h. Then, the implants were decalcified, embedded and sliced for haematoxylin and eosin (H&E) staining, and the fat vacuoles were stained with a Perilipin-1 antibody (1:100, Cell Signaling Technology Cat# 9349) for immunohistochemistry. All data were calculated and analysed with ImageJ software.

### H&E staining, Masson’s trichrome staining, and immunohistochemical staining

The sections were removed from the embedding agent, placed in xylene, and then rehydrated with decreasing concentrations of ethanol. For H&E staining, the sections were placed in a haematoxylin dye solution for 8–10 min and then stained with an eosin solution for 8–10 min after differentiation with differentiation solution (1% HCl and 70% alcohol). A Masson’s trichrome staining kit (Sigma, USA) was used, and staining was performed according to the manufacturer’s protocol. For immunohistochemistry, after deparaffinization and hydration, the sections were treated with H_2_O_2_ for 30 min to block endogenous peroxidase activity and treated with trypsin and pepsin to retrieve the antigens. Next, the sections were blocked with 3% BSA for 30 min and then incubated with the primary antibody at 4 °C overnight. Afterwards, the sections were incubated with a secondary antibody (HRP-labelled) and finally visualized with DAB through a chromogenic reaction. After nuclear counterstaining, dehydration and mounting, the stained sections were observed and photographed under a light microscope (Leica, Germany).

### Adipogenic differentiation of hMSCs in vitro

hMSCs were cultured in adipogenic differentiation medium (10% FBS DMEM, 1 μM dexamethasone, 10 μg/ml insulin, 0.5 mM IBMX, 0.2 mM indomethacin, and 100 IU/ml penicillin–streptomycin) in 12-well plates at a density of 6 × 10^4^ cells/well. Every 3 days, the adipogenic differentiation medium was replaced until the cells were used in subsequent experiments.

### Oil red O (ORO) staining

After three washes with PBS, hMSCs were fixed with 4% paraformaldehyde for 25 min. hMSCs were washed again with PBS three times and then stained with ORO working solution (2 parts of ddH_2_O and 3 parts of 0.5 g of ORO powder in 100 ml of isopropanol) at room temperature. The working solution was removed, and nonspecifically bound dye was removed by rinsing with PBS. The stained hMSCs were observed and photographed under a microscope. For quantification of the staining, isopropanol was added to extract ORO, and the absorbance (520 nm) of a 200 μl aliquot was measured in a 96-well plate.

### Transmission electron microscopy (TEM)

hMSCs were harvested, centrifuged, and then fixed with 4% formaldehyde and 2.5% glutaraldehyde in 0.1 M PB (pH 7.4) overnight. After the samples were pre-embedded in agarose, they were postfixed with 1% OsO_4_ in 0.1 M phosphate buffer (pH 7.0) for 2 h at room temperature. Samples were dehydrated at room temperature with increasing concentrations of ethanol (30%, 50%, 70%, 80%, 95% and 100%) for 20 min and finally incubated with two acetone solutions for 15 min. After resin penetration, polymerization, ultrathin sectioning and staining, the ultrastructure of hMSCs was observed and photographed with TEM.

### Immunofluorescence staining

hMSCs were seeded onto confocal dishes or sterile glass coverslips. After different treatments, the cells were fixed with 4% paraformaldehyde for 25 min and permeabilized with 0.1% Triton X-100 for 15 min. Then, the cells were blocked with 10% goat serum for 30 min and incubated with anti-APPL1 (1:200, Santa Cruz Biotechnology Cat# sc-271901), anti-MYOF (1:100, Abcam Cat# ab178386), anti-LAMP2 (1:100, Abcam Cat# ab25631) or anti-Galectin 3 (1:100, Abcam Cat# ab209344) primary antibodies overnight at 4 °C. Next, anti-rabbit (1:500, Cell Signaling Technology Cat# 2975) or anti-mouse (1:500, Cell Signaling Technology Cat# 4409) fluorophore-labelled secondary antibodies were incubated with the cells at room temperature for 1 h. Nuclei were stained with 4,6-diamidino-2-phenylindole (DAPI). Finally, the fluorescence staining was observed and photographed with a confocal microscope (LSM880, Carl Zeiss). The images were obtained from the confocal imaging system and analysed with ImageJ software.

### LysoTracker staining

The lysosomes were observed by staining with LysoTracker Red (Beyotime, Shanghai, China) according to the manufacturer’s protocol. Briefly, the medium of hMSCs was replaced, and the cells were coincubated with the LysoTracker Red working solution (1 μl of LysoTracker Red was mixed with 20 ml of complete medium) and Hoechst 33342 to stain the nuclei at 37 °C for 30 min. Then, the working solution was discarded, and fresh cell medium was added. For experiments requiring exposure to LLOMe, medium containing 1 mM LLOMe was added to the plate for 5 min, 15 min or 30 min. Finally, the fluorescence staining of lysosomes was observed and photographed with a confocal microscope (LSM880, Carl Zeiss). The images were obtained from the confocal imaging system and analysed with ImageJ software.

### Construction and treatment of the mouse osteoporosis model

All C57BL/6J mice were obtained from the Laboratory Animal Center of Sun Yat-Sen University (Animal ethical approval No. 2021d184) at the age of 6–8 weeks and housed under pathogen-free conditions on a 12-h light–dark cycle with free access to water and food throughout the experiment. After the mice were 16 months of age, they were used for a related study as a model of senile osteoporosis. Mouse models of postmenopausal osteoporosis were constructed using female C57BL/6J mice at the age of 8 weeks. Ovariectomy was performed on the mice, and the mice that underwent sham surgery were used as the control group. Mouse models of postmenopausal osteoporosis were used to confirm the therapeutic effect of APPL1 on osteoporosis. Briefly, 7 days after ovariectomy, the mice were injected with the APPL1 overexpression adenovirus through the tail vein. After 3 months, the mice were sacrificed, and the femora of the mice were subjected to microcomputed tomography (Micro-CT), H&E staining and immunohistochemical staining to analyse the bone mass.

### Micro-CT

The femora isolated from C57BL/6J mice were fixed with 4% polyoxymethylene for 2 days. A Micro-CT system (Siemens) was used for scanning and analysis. Briefly, the full-length femora were scanned, and the region below the lower growth plate of approximately 50 mm for 100 slices was selected as the region of interest. According to the protocol in our previous study [31], images a voltage of 80 kV, a current of 500 lA, an effective pixel size of 8.82 lm and an exposure time of 1500 ms were taken in each of the 360 rotating steps to examine trabecular bone. The two-dimensional and three-dimensional structures were produced by reconstructing image slices using Micro-CT analysis software. Bone morphometry was analysed by determining the bone volume/total volume (BV/TV), trabecular thickness (Tb.Th), cortical thickness (Ct.Th), trabecular number (Tb.N) and trabecular spacing (Tb.Sp).

### Coimmunoprecipitation (Co-IP) and LC–MS/MS

Co-IP was performed with a Dynabeads™ Protein G Immunoprecipitation Kit (Invitrogen). According to the manufacturer’s protocol, the cells were lysed with IP lysis buffer containing a 1% phosphatase and protease inhibitor cocktail on ice for 30 min, and the supernatant was collected after centrifugation at 14,000×*g* for 15 min. The samples were precleared with magnetic beads to eliminate nonspecific binding. Then, the required amount of primary antibody (anti-APPL1, MYOF, or DDDDK-Tag) was added to the precleared samples and incubated overnight at 4 °C with moderate rotation. Afterwards, magnetic beads were added to the samples and incubated for 2 h. The magnetic beads were then collected and washed 3 times with wash buffer. The magnetic beads were mixed with SDS–PAGE loading buffer and boiled for 10 min at 100 °C. Immunoprecipitates were collected after the magnetic beads had been removed. Samples were resolved on SDS–PAGE gels, which were then dyed with a Coomassie blue staining kit (Beyotime Institute of Biotechnology). The APPL1-interacting proteins in hMSCs were collected for LC–MS/MS analysis, and the raw data are shown in the supplementary materials. The endogenous interaction between the two proteins in hMSCs was confirmed using Western blotting. The specially designed plasmids containing different protein domains were transfected into 293 T cells. After 48 h, proteins extracted from the cells were used to confirm the interaction with the structural domain of APPL1.

### Statistical analysis

The data in our study were analysed using GraphPad Prism 8 (San Diego, CA, USA) and SPSS 22.0 software (Chicago, IL, USA). All results are presented as the means ± standard deviations (SD). Significant differences between two groups were analysed using Student’s *t* test, differences among more than two groups were analysed using one-way or two-way analysis of variance (ANOVA), and the correlation analysis was conducted by calculating Pearson’s correlation coefficient. Significance was established at *P* < 0.05.

### Role of funders

The funders had no role in the study design, data collection, data analysis, interpretation, writing of the manuscript, or decision to submit the paper for publication.

## Results

### Downregulation of APPL1 expression in osteoporosis

**Fig. 1:**
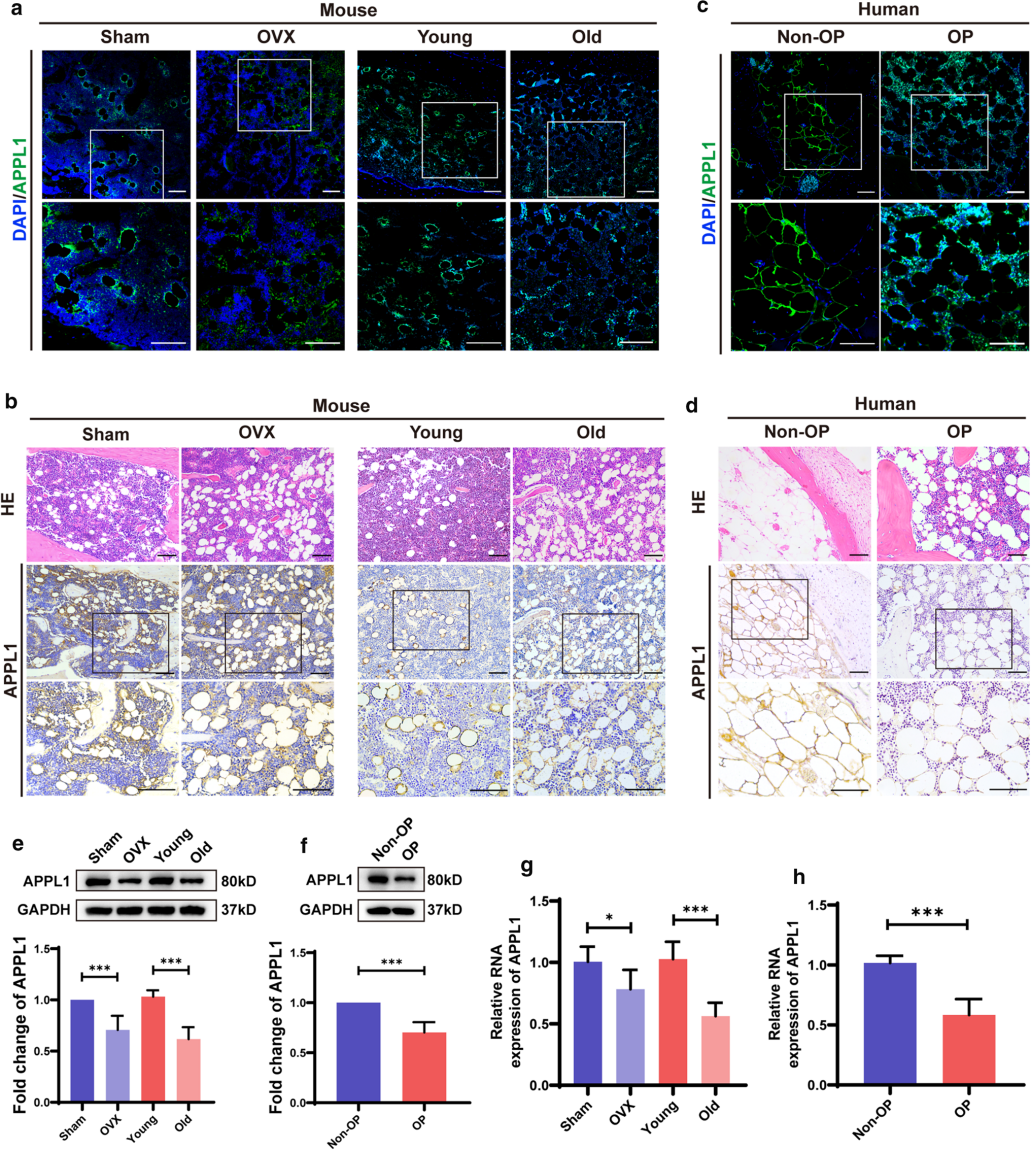
APPL1 expression is downregulated in individuals with osteoporosis. **a** Immunofluorescence and **b** immunohistochemical staining for APPL1 in the femurs of C57BL/6J mice that underwent sham surgery, OVX mice, young mice and elderly mice. **c** Immunofluorescence and **d** immunohistochemical staining for APPL1 in the femoral heads of nonosteoporotic patients (Non-OP) and patients with osteoporosis (OP). **e**, **g** The APPL1 protein and mRNA levels in bone marrow MSCs from C57BL/6J mice that underwent sham surgery, OVX mice, young mice and elderly mice were detected using Western blotting and qRT–PCR, respectively. **f**, **h** Western blotting and qRT–PCR were used to detect the APPL1 protein and mRNA levels, respectively, in hMSCs from nonosteoporotic patients and patients with osteoporosis. Scale bar = 100 μm. All data are presented as the means ± SD, *n* = 6 per group. Statistical differences were determined using Student’s *t* test. *ns* not statistically significant, *P < 0.05, **P < 0.01, and ***P < 0.001

We generated senile osteoporosis and postmenopausal osteoporosis mouse models as mouse osteoporosis models, and human osteoporosis samples were collected from clinical patients to investigate the pathogenic mechanisms of osteoporosis. Patients with osteoporosis were recruited from individuals with postmenopausal osteoporosis, and healthy controls were recruited from individuals who suffered accidents. The postmenopausal osteoporosis model was constructed by performing an ovariectomy (OVX) on 8-week-old C57BL/6J mice and treating the same number of 8-week-old C57BL/6J mice with a sham surgery as controls. Eighteen-month-old C57BL/6J mice were directly used as the elderly osteoporosis model, while 8-week-old C57BL/6J mice served as controls. The expression level in the human osteoporosis bone sample was downregulated compared with that in the nonosteoporosis sample based on immunohistochemistry and immunofluorescence staining. Furthermore, the APPL1 expression level in tissues from the mice with postmenopausal osteoporosis and elderly osteoporosis was significantly decreased compared with that in mouse bone tissue slices from the sham surgery and young groups, as shown by immunohistochemistry and immunofluorescence staining (Fig. 1a–d). We isolated primary MSCs from osteoporotic and corresponding control bone marrow to further investigate the APPL1 level in bone marrow MSCs, and the APPL1 mRNA and protein levels were significantly downregulated in the osteoporosis group compared with the control group (Fig. 1e–h), consistent with the results obtained from bone tissue sections. By comparing APPL1 expression levels between the control group and osteoporosis group, we hypothesized that the occurrence and development of osteoporosis are closely related to the downregulation of APPL1 expression.

### Decreasing APPL1 expression promotes hMSCs adipogenic differentiation in vitro

**Fig. 2:**
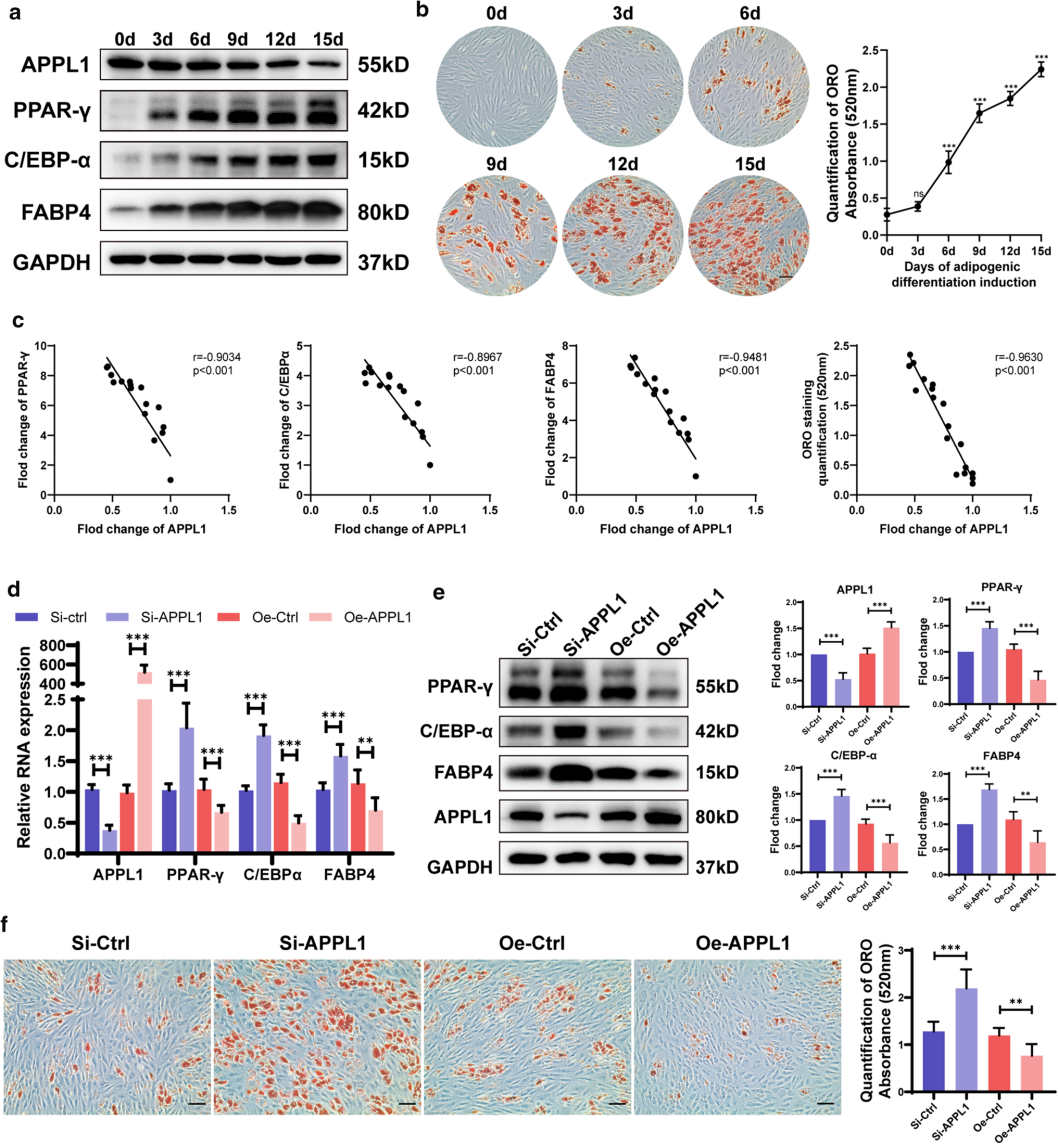
Decreasing APPL1 expression promoted the adipogenic differentiation of hMSCs in vitro. **a** The Protein levels of adipogenic markers PPAR-γ, C/EBP-α, and FABP4 along with the day of hMSC adipogenic differentiation. **b** ORO staining and quantification along with the day of hMSC adipogenic differentiation. **c** Pearson correlation analysis revealed the correlation between APPL1 expression and ORO staining quantification, PPAR-γ, C/EBP-α, and FABP4 levels during hMSC adipogenic differentiation. **d** Relative RNA expression of the adipogenic markers PPAR-γ, C/EBP-α, and FABP4 were detected using qRT–PCR. **e** Protein levels of the adipogenic markers PPAR-γ, C/EBP-α, and FABP4 were detected by using Western blot analysis. Quantification of the data is shown in the right panel. **f** SiRNAs and APPL1 overexpression lentiviruses were transfected into hMSCs, and cells were then cultured in adipogenic medium. ORO staining and quantification on day 10. Scale bar = 50 μm. All data are presented as the means ± SD, *n* = 3 per group in (**a**, **b**), *n* = 15 in (**c**), *n* = 6 per group in (**d**–**f**). Statistical differences were determined using Student’s *t* test or ANOVA. *ns* not statistically significant, *P < 0.05, **P < 0.01, and ***P < 0.001

Osteoporosis is usually accompanied by an increase in bone marrow adipose tissue. Several studies have shown that changes in hMSCs adipose differentiation are closely related to the initiation and progression of osteoporosis. Therefore, we explored the variation tendency of APPL1 expression during adipogenic differentiation of hMSCs. The results showed that APPL1 was downregulated along with the induction of adipogenesis, as shown by Western blotting and ORO staining (Fig. 2a, b). A correlation analysis between APPL1 levels and the expression of markers of adipogenic differentiation (PPARγ, CEBPα, and FABP4), and ORO staining quantification was performed to show the relationship between APPL1 and hMSCs adipogenic differentiation, and the results revealed that the degree of APPL1 expression was negatively correlated with the expression of adipogenic differentiation markers and ORO quantification (Fig. 2c).

To explore the effect of APPL1 on hMSC adipogenic differentiation, we synthesized an APPL1 knockdown siRNA and overexpression lentivirus to downregulate and overexpress APPL1 in hMSCs, respectively. Using qRT–PCR and Western blotting detection, the mRNA and protein levels of adipogenic differentiation markers in hMSCs were found to be substantially increased with APPL1 knockdown (Fig. 2d, e), indicating that the adipogenic differentiation capacity of hMSCs was significantly increased when the APPL1 levels were decreased. Similarly, ORO staining showed that the formation of adipocytes was significantly increased following APPL1 knockdown (Fig. 2f). In contrast to the knockdown experiment, APPL1 was overexpressed by transfecting the APPL1 overexpression lentivirus, and the expression of adipogenic differentiation markers was decreased, as evidenced by qRT–PCR and Western blotting (Fig. 2g, h). The number of lipid droplets in hMSCs was also significantly reduced, as shown by ORO staining (Fig. 2f). These results indicate that APPL1 exerted a negative regulatory effect on hMSC adipogenic differentiation in vitro.

### Decreasing APPL1 expression promotes the adipogenic differentiation of hMSCs in vivo

**Fig. 3:**
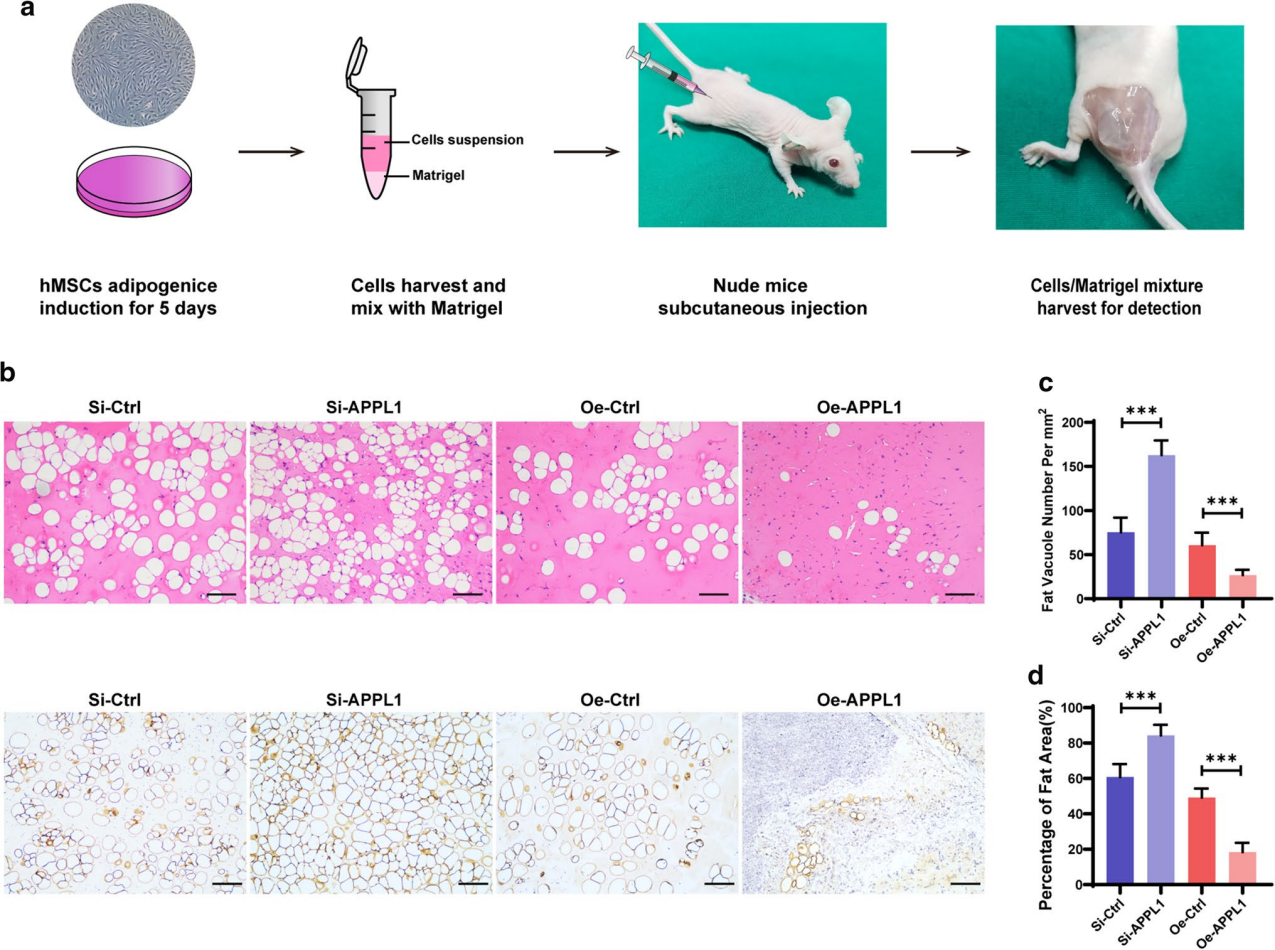
Decreasing APPL1 expression promoted the adipogenic differentiation of hMSCs in vivo. **a** Schematic diagram of the hMSCs adipogenic differentiation experiment in vivo. **b** H&E staining and Immunohistochemistry showing the fat vacuoles in the APPL1 knockdown group and overexpression group. **c** Quantification of fat vacuole numbers in hMSCs adipogenic differentiation experiments in vivo. **d** Quantification of the fat area percentage in hMSCs adipogenic differentiation experiment in vivo. Scale bar = 100 μm. All data are presented as the means ± SD, *n* = 6 per group. Statistical differences were determined using Student’s *t* test. *ns* not statistically significant, *P < 0.05, **P < 0.01, and ***P < 0.001

We performed a mature human stem cell culture experiment in vivo to assess the effect of APPL1 on hMSCs adipogenic differentiation in vivo. The experiments were modified based on the methods reported by Khan ZA [30]. Briefly, after APPL1 knockdown and overexpression, the hMSCs were mixed with Matrigel, injected subcutaneously into nude mice, and analysed after 6–8 weeks. Subsequently, the cells/Matrigel implanted into the subcutaneous tissue were harvested, and prepared into paraffin slices, and the level of adipogenic differentiation was evaluated (Fig. 3a). The number of adipocytes was quantified using H&E staining. The number of adipocytes increased substantially after APPL1 knockdown and decreased significantly after APPL1 overexpression. This finding was further supported by immunohistochemical staining for the adipocyte marker Perilipin-1 (Fig. 3b–d). These discoveries were consistent with the results of the hMSCs culture experiment in vitro, confirming that APPL1 exerts a negative effect on regulating hMSCs adipogenic differentiation in vivo.

### APPL1 binds to MYOF and inhibits MYOF degradation by the ubiquitination-protease pathway

**Fig. 4:**
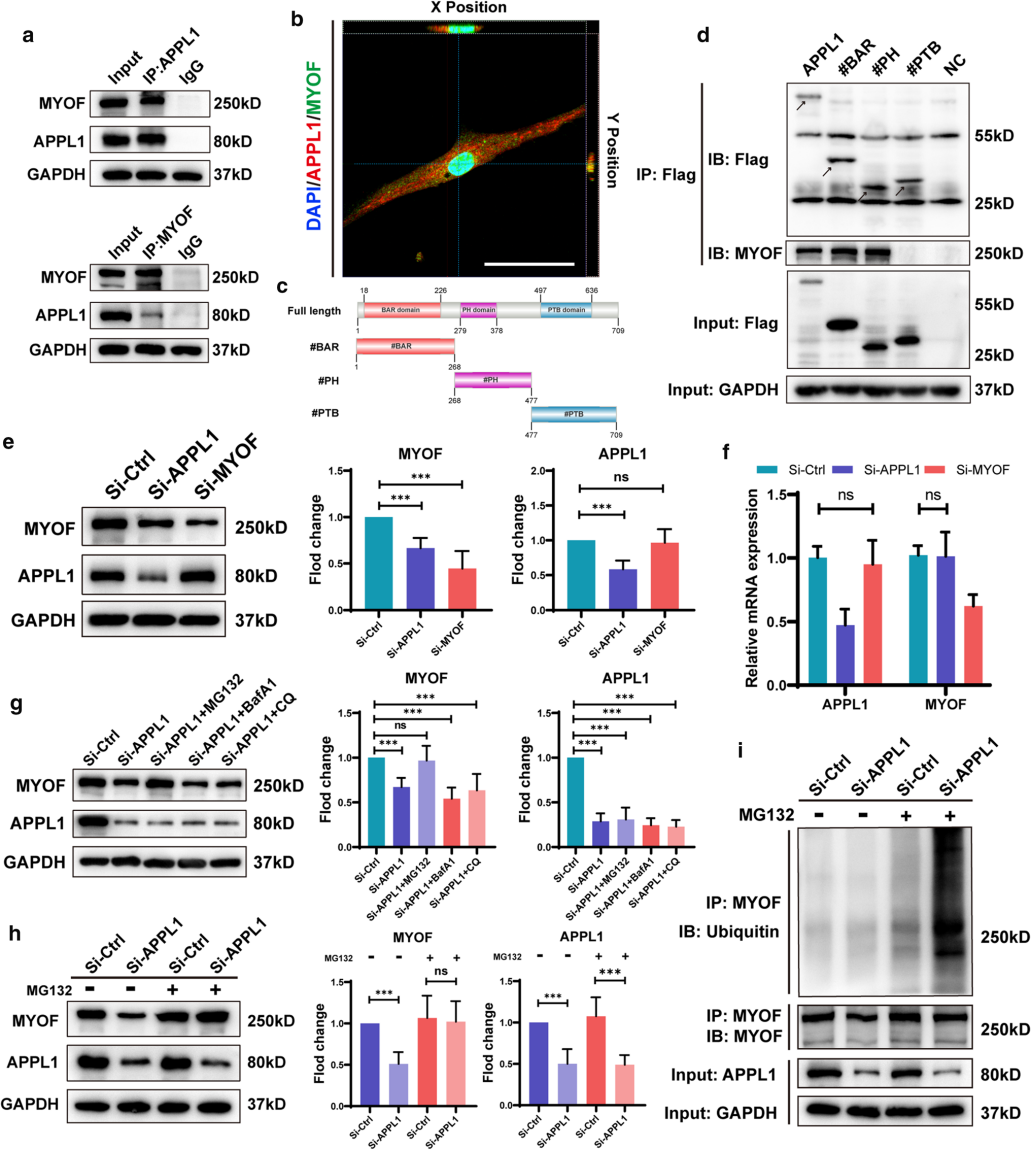
APPL1 binds to MYOF and inhibits MYOF degradation by the ubiquitination-protease pathway. **a** hMSCs lysates were immunoprecipitated with MYOF, APPL1 or IgG antibodies. The endogenous interactions between the APPL1 and MYOF proteins in hMSCs were detected using Western blot analysis. **b** Immunofluorescence staining revealed the colocalization of APPL1 (red) and MYOF (green), and the nucleus was stained with DAIP (blue). **c** Diagram showing the structural domains and sequence of APPL1. **d** The binding sites of APPL1 that interacted with MYOF were detected using Western blot analysis. **e** Changes in protein levels after siRNA intervention were detected using Western blot analysis. Quantification of the data is shown in the right panel. **f** APPL1 and MYOF mRNA levels were not different between the APPL1 knockdown group and the MYOF knockdown group in the qRT–PCR analysis. **g** Western blot analysis of MYOF protein levels in the siControl group or APPL1 knockdown group treated with lysosome inhibitors (BafA1, 10 μM; CQ, 10 mM) and the proteasome inhibitor (MG132, 10 μM). Quantification of the data is shown in the right panel. **h** Western blot analysis showed whether proteasome inhibitors MG132 could reverse MYOF protein degradation during APPL1 knockdown. Quantification of the data is shown in the right panel. **i** Immunoprecipitation showed the levels of MYOF ubiquitination, and the control group and APPL1 knockdown group were treated with or without MG132. Scale bar = 50 μm. All data are presented as the means ± SD, *n* = 6 per group. Statistical differences were determined using Student’s *t* test or ANOVA. *ns* not statistically significant, *P < 0.05, **P < 0.01, and ***P < 0.001

To explore the mechanism by which APPL1 regulates adipogenic differentiation, we performed co-IP and LC–MS/MS to detect downstream proteins that bind to APPL1. The mass spectrometry results indicated that many proteins interacted with APPL1, as shown in the supplementary materials. MYOF was detected in the co-IP samples, indicating that MYOF interacted with APPL1. Next, we further tested the binding of endogenous APPL1 and MYOF in hMSCs by performing a co-IP assay, and the results showed that endogenous APPL1 and MYOF in hMSCs bound to each other, consistent with the LC–MS/MS results (Fig. 4a).

In addition, immunofluorescence staining was used to clarify the intracellular binding and location of APPL1 and MYOF. The fluorescence colocalization analysis revealed that APPL1 and MYOF showed close colocalization. Both APPL1 and MYOF were expressed in the nucleus and cytoplasm; however, they mainly interacted in the cytoplasm (Fig. 4b).

To further determine the domain of APPL1 that binds MYOF, we constructed plasmids with three different domains of APPL1 (Fig. 4c). After transfection into 293 T cells, the co-IP results confirmed that APPL1 interacted with MYOF (Fig. 4d) through its BAR and PH domains.

Then, we explored the relationship between APPL1 and MYOF. First, SiRNA interference was performed on APPL1 and MYOF, and the expression levels were measured with Western blotting. The APPL1 levels did not change significantly following MYOF knockdown, whereas the MYOF levels decreased significantly after APPL1 knockdown (Fig. 4e). To verify whether the change in protein levels was driven by the change in mRNA levels, qRT-PCR assays were performed, and the results showed that the APPL1 and MYOF mRNA levels did not change (Fig. 4f), indicating that APPL1 regulates MYOF expression at the protein level.

The proteasome and lysosomal pathways are classic intracellular protein degradation pathways. We treated APPL1 knockdown hMSCs with proteasome inhibitors MG132 and the lysosome inhibitors Chloroquine (CQ) and Bafilomycin A1 (BafA1) to investigate the mechanism by which APPL1 modulates MYOF protein levels. The MYOF level in APPL1 knockdown hMSCs was restored to baseline levels compared with the control group after treatment with the proteasome inhibitor MG132. In contrast, the MYOF levels were not altered significantly after treatment with lysosome inhibitors (CQ and BafA1) (Fig. 4g, h), indicating that APPL1 regulates MYOF levels by affecting proteasome degradation pathways.

The MYOF protein was enriched in hMSCs, as shown by immunoprecipitation assays, and the level of ubiquitinated MYOF was detected using Western blotting (Fig. 4i). In the absence of the proteasome inhibitor MG132, the level of MYOF ubiquitination was low in both the control group and the APPL1 knockdown group, indicating that the ubiquitination level in cells was affected by proteasomal degradation; therefore, MG132 was added to determine the degree of ubiquitination. Compared with the control group, the level of MYOF ubiquitination increased in the APPL1 knockdown group following the addition of MG132, indicating that APPL1 binding to MYOF influenced the proteasomal degradation of MYOF by modulating the MYOF ubiquitination level.

### Downregulation of MYOF expression causes lysosomal dysfunction during the adipogenic differentiation of hMSCs

**Fig. 5:**
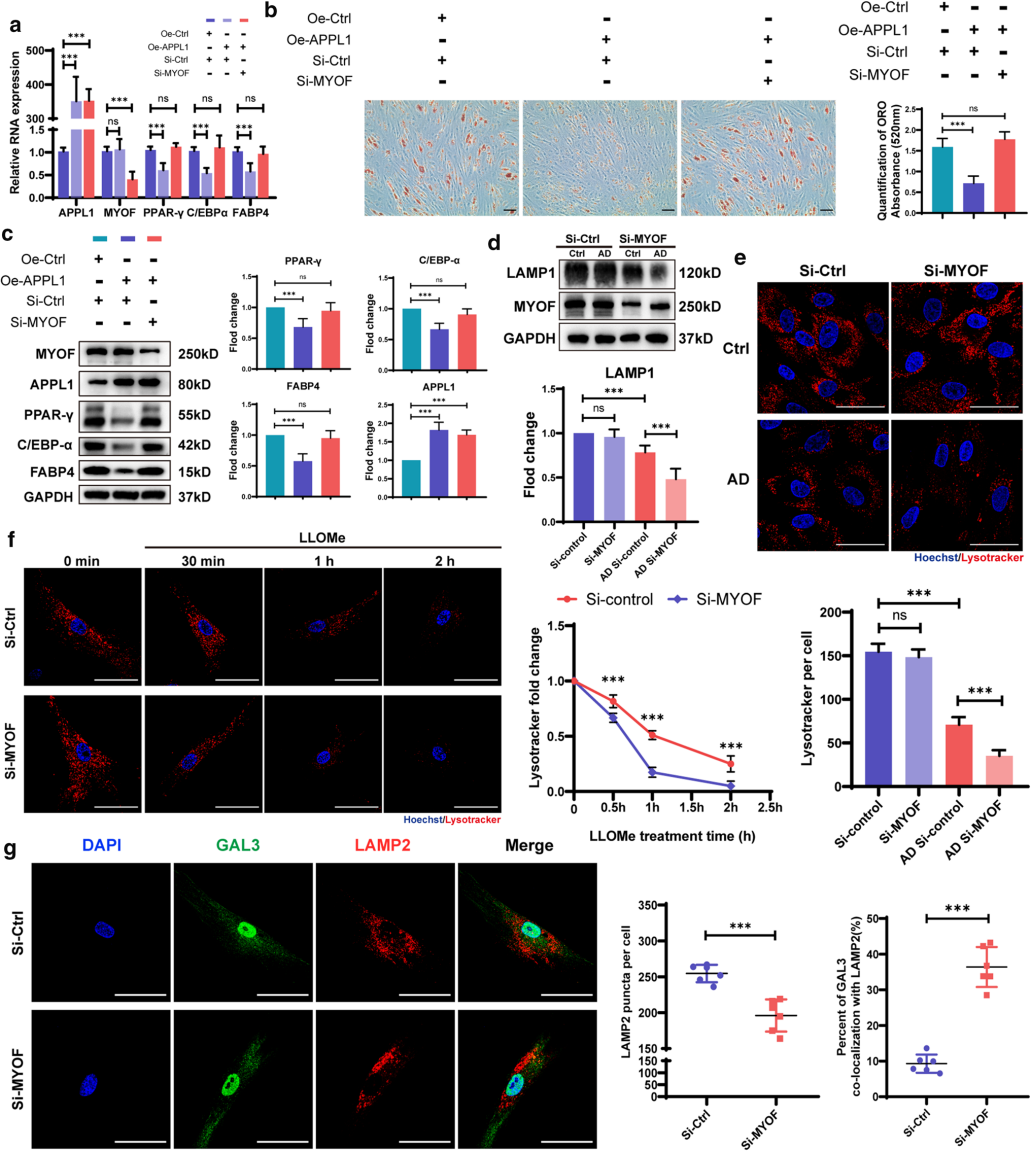
Downregulated MYOF expression causes lysosomal dysfunction during the adipogenic differentiation of hMSCs. **a** Relative mRNA expression of adipogenic markers was detected using qRT–PCR after APPL1 overexpression or MYOF knockdown. **b** hMSCs adipogenesis was revealed with ORO staining and quantification after APPL1 overexpression or MYOF knockdown. **c** Protein levels of adipogenic markers were detected using Western blot analysis after APPL1 overexpression and MYOF knockdown. Quantification of the data is shown in the right panel. **d** After MYOF knockdown, the protein levels of lysosome markers (LAMP1) were analysed using Western blotting. Quantification of the data is shown in the lower panel. **e** The lysosomes of hMSCs with or without MYOF knockdown that were cultured in the control or adipogenic induction medium were stained with LysoTracker and revealed by immunofluorescence staining. Quantification of LysoTracker is shown in the lower panel. **f** Time course of LysoTracker staining in siControl and MYOF knockdown hMSCs following treatment with LLOMe for 0, 0.5, 1 and 2 h. Quantification of LysoTracker is shown in the right panel. **g** Immunofluorescence staining for GAL3 (green) and LAMP2 (red) along with adipogenic induction, and the nucleus was stained with DAIP (blue). Quantification of the data is shown in the right panel. Scale bar = 50 μm. All data are presented as the means ± SD, n = 6 per group. Statistical differences were determined using Student’s *t* test or ANOVA. *ns* not statistically significant, *P < 0.05, **P < 0.01, and ***P < 0.001

As described above, APPL1 exerts a negative regulatory effect on hMSCs adipogenic differentiation, and MYOF is a downstream protein of APPL1. We next explored the role and relationship between APPL1 and MYOF in adipogenic differentiation by performing APPL1 overexpression and MYOF knockdown experiments. MYOF knockdown reversed the negative regulatory effect of APPL1 on hMSCs adipogenic differentiation, suggesting that MYOF is a key downstream molecule by which APPL1 negatively modulates hMSCs adipogenic differentiation (Fig. 5a–c).

The experiments described above showed that APPL1 acts through MYOF, but more research is necessary to confirm how MYOF, the downstream protein of APPL1, affects hMSCs adipogenic differentiation. MYOF, a member of the Ferlin family, has been proven to function in the membrane repair system, and reduced levels of MYOF on the lysosomal membrane result in excess lysosomal stress during adipogenic differentiation. Therefore, we proposed that the increased adipogenic differentiation of hMSCs results from lysosomal degradation and loss due to reduced MYOF expression on lysosome membranes driven by the lack of APPL1. To confirm this hypothesis, we used LysoTracker to label lysosomes in hMSCs undergoing adipogenic differentiation after MYOF knockdown. The results of the Western blot assessment of the lysosomal marker lysosomal membrane protein 1 (LAMP1) indicated that the loss of MYOF during hMSC adipogenic differentiation resulted in a decrease in lysosome number (Fig. 5d). Fluorescence signals revealed that the number of lysosomes was significantly reduced after MYOF knockdown compared with that of the control group during adipogenic differentiation (Fig. 5e), which was consistent with the Western blotting results. Furthermore, the finding that the MYOF knockdown group did not show a significant difference in the number of lysosomes compared with the control group without the induction of adipogenic differentiation differed from the result observed in the adipogenic differentiation group. We propose that this difference is due to different workloads in lysosomes between adipogenic hMSCs and nonadipogenic hMSCs, as hMSCs adipogenic differentiation requires lysosomes to coordinate various related functions, resulting in lysosome overload and increased susceptibility to deterioration and dysfunction. Therefore, the lack of MYOF, which is involved in maintaining lysosomal membrane stability, likely resulted in a decrease in the number of lysosomes under overload conditions.

We then investigated the function of MYOF in stabilizing the lysosomal membrane in hMSCs by inducing lysosomal stress through the application of the lysosomal stress inducer LLOMe, and the results showed that the number of lysosomes decreased faster in the MYOF-deficient group due to lysosomal stress (Fig. 5f). Furthermore, the aggregation of the lysosomal damage marker Galectins-cytoplasmic proteins 3 (GLA3) in lysosomes was obviously increased in MYOF-deficient hMSCs subjected to lysosomal stress (Fig. 5g), indicating that MYOF deficiency leads to decreased lysosomal stability and an increased risk of damage under overload.

### MYOF deficiency in hMSCs inhibits autophagy flux by altering lysosomal function

**Fig. 6:**
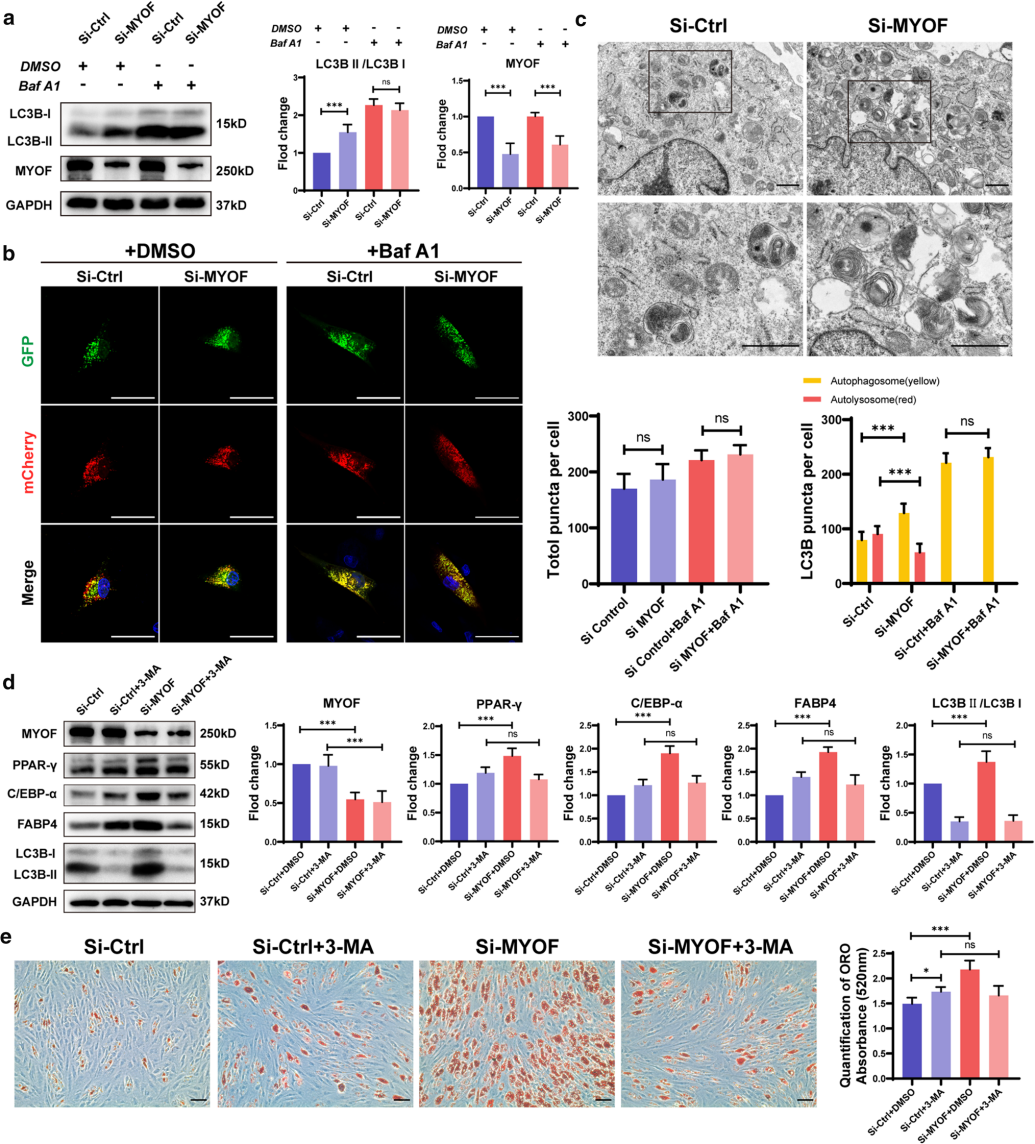
MYOF deficiency in hMSCs inhibits autophagy flux by altering lysosomal function. **a** Western blot analysis of autophagy flux and autophagy activation after MYOF knockdown under adipogenic induction conditions with or without BafA1. **b** hMSCs were infected with a GFP-mCherry-LC3B lentivirus for 24 h, and fluorescent staining was detected using confocal microscopy after MYOF knockdown. **c** Transmission electron microscopy (TEM) was used to reveal autophagosomes in hMSCs under adipogenic induction conditions. **d** Protein levels of LC3B and adipogenic differentiation markers were evaluated using Western blotting after transfection with siControl and siMYOF and treatment with or without 3-MA. **e** ORO staining and quantification were performed to reveal adipogenesis after transfection with siControl and siMYOF and treatment with or without 3-MA. **b**, **e** Scale bar = 50 μm. **c** Scale bar = 1 μm. All data are presented as the means ± SD, *n* = 6 per group. Statistical differences were determined using Student’s *t* test or ANOVA. *ns* not statistically significant, *P < 0.05, **P < 0.01, and ***P < 0.001

Lysosomes, as important organelles in cells, play an important role in maintaining normal physiological functions, particularly as an important part of the autophagy–lysosome pathway. After MYOF knockdown in hMSCs during adipogenic differentiation, we further studied the change in autophagy to explore the effect of lysosomal dysfunction caused by MYOF knockdown in hMSCs. The level of LC3BII and the LC3BII/LC3BI ratio are closely related to the level of autophagy. During the activation of autophagy, LC3BI is gradually transformed into LC3BII, and the proportion of LC3BII increases. After MYOF knockdown, the Western blotting results revealed an increase in the LC3BII/LC3BI ratio, indicating a significant increase in the number of autophagosomes in hMSCs (Fig. 6a). However, we were unclear whether MYOF knockdown truly led to the activation of autophagy or inhibited autophagy flux by blocking autophagosome degradation. Therefore, the lysosomal inhibitor BafA1, which effectively inhibits the proton pump and destroys the acidic environment in lysosomes such that lysosomes do not degrade autophagosomes normally, was added to both the control and knockout groups to determine whether autophagy was activated. The results showed no significant difference in the LC3BII/LC3BI ratio between the control group and MYOF knockdown group after addition of the lysosomal inhibitor BafA1 (Fig. 6a), implying that the increase in LC3BII levels after MYOF knockdown was primarily caused by the suppression of autophagosome degradation. Specifically, autophagy flux was blocked due to the lack of lysosomes to subsequently degrade autophagosomes by forming autolysosomes, and autophagosomes finally aggregated in hMSCs.

We confirmed the conclusions described above by constructing a GFP-mCherry-LC3B double label overexpression lentivirus to observe autophagy flux in hMSCs. The LC3B protein was linked to two different fluorescent proteins, the green fluorescence protein (GFP) and mCherry protein, and fluorescence quenching of GFP occurred in the lysosomal acidic environment. Therefore, autophagosomes were observed as yellow fluorescence indicating colocalization of GFP and mCherry fluorescent proteins, and autophagolysosomes, which are autophagosomes fused with lysosomes, were observed as red fluorescence, indicating mCherry fluorescence protein. Confocal microscopy revealed that after MYOF knockdown in hMSCs, the amount of red fluorescence decreased, while the amount of yellow fluorescence increased (Fig. 6b). No difference in the amount of yellow fluorescence was observed between the knockdown groups and control groups after BafA1 treatment, confirming that the increase in the number of autophagosomes was not induced by enhanced autophagy activation but rather was due to decreased degradation caused by the reduction in the number of lysosomes. Furthermore, using electron microscopy, we observed that a large number of autophagosomes with double membrane structures accumulated in the visual field, while few autophagolysosomes were observed in MYOF knockdown hMSCs (Fig. 6c). Overall, MYOF deficiency reduces the number and function of lysosomes in hMSCs, resulting in dysfunctional autophagic degradation, suppression of autophagy flux, and dysfunction of intracellular protein degradation in hMSCs during adipogenic differentiation.

Previous studies have documented an important role for autophagy in the balance of hMSCs differentiation, and we believe that inhibiting autophagy flux and autophagosome overload when autophagosome degradation is suppressed are the main factors explaining the increase in the adipogenic differentiation capacity of APPL1-deficient hMSCs. We applied the autophagy inhibitor 3-methyladenine (3-MA) to suppress autophagosome formation and reveal the relationship between autophagosome accumulation and the adipogenic differentiation capacity of hMSCs. The results showed that adipogenic differentiation returned to normal levels when autophagosome overload was relieved by 3-MA compared with that of the control group, as shown by Western blotting and ORO staining (Fig. 6d, e).

### Overexpression of APPL1 relieves osteoporosis in mice

**Fig. 7:**
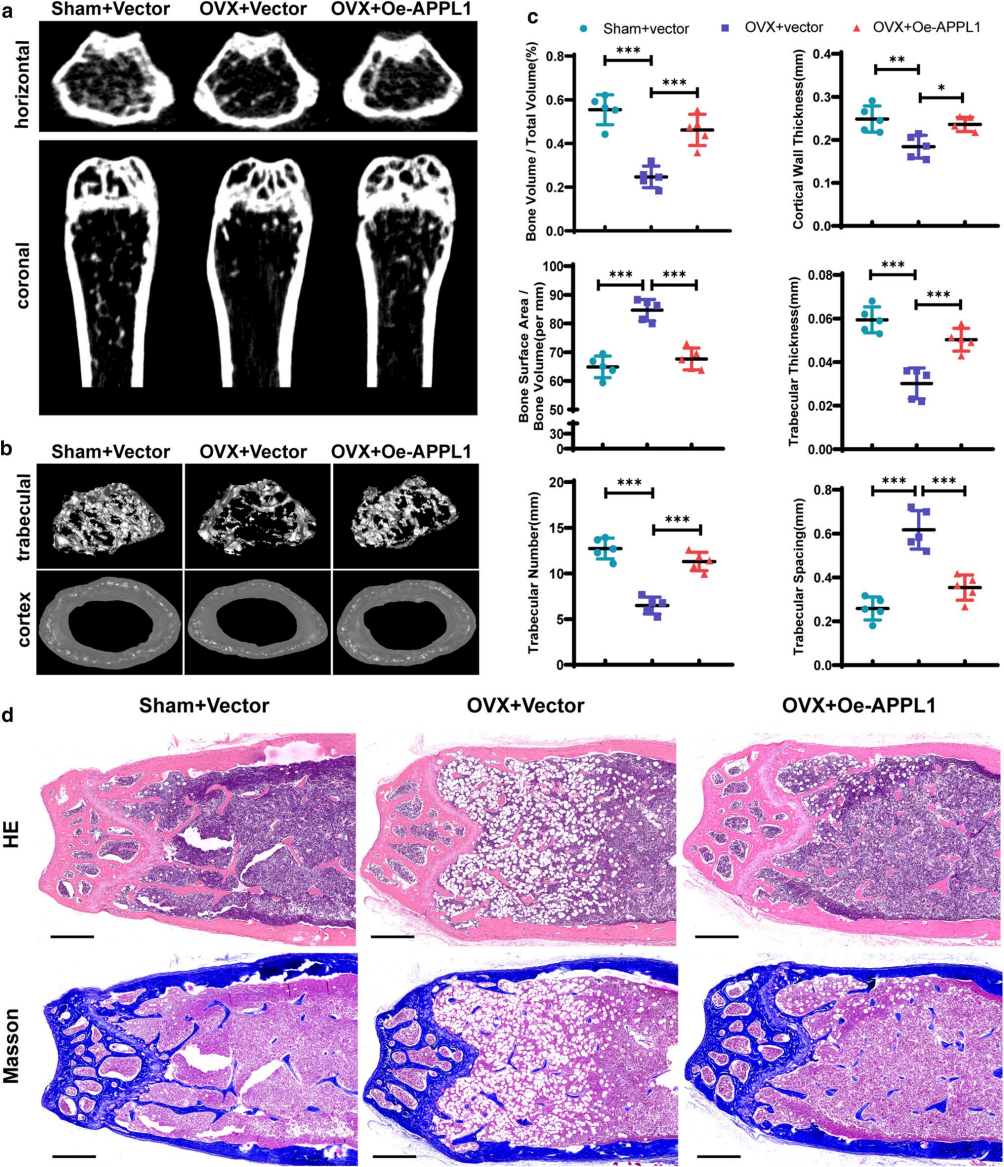
Overexpression of APPL1 relieves osteoporosis in mice. **a** Coronal and horizontal images of femurs in mice in the control group, mice with postmenopausal osteoporosis or APPL1-overexpressing adenovirus-treated mice were captured using micro-CT. **b** Three-dimensional micro-CT reconstruction was used to analyse cortical bone and trabecular bone. **c** BV/TV, BA/BV, trabecular thickness, trabecular number, trabecular spacing and cortical wall thickness were analysed in the control group of mice, mice with postmenopausal osteoporosis or APPL1-overexpressing adenovirus-treated mice. **d** H&E staining and Masson’s trichrome staining of tissues from the control group of mice, mice with postmenopausal osteoporosis or APPL1-overexpressing adenovirus-treated mice. Scale bar = 500 μm. All data are presented as the means ± SD, *n* = 6 per group. Statistical differences were determined using ANOVA. *ns* not statistically significant, *P < 0.05, **P < 0.01, and ***P < 0.001

Our previous studies have shown that APPL1 deficiency occurs in osteoporosis and that APPL1 plays a critical role in regulating hMSCs adipogenic differentiation. To explore the potential of APPL1 to target osteoporosis, we constructed a mouse model of osteoporosis to investigate whether the progression of osteoporosis would be halted by modifying APPL1 levels in mice. Because the elderly osteoporosis model has a long construction period, an assessment of treatment efficacy is difficult; therefore, a murine postmenopausal osteoporosis model was used in our studies to examine the effect of APPL1 on osteoporosis. An adenovirus overexpressing the full-length APPL1 sequence was designed, constructed, and injected into C57 mice via the tail vein 1 week after ovariectomy or sham surgery. The femurs were removed for Micro-CT scanning and tissue sectioning 8 weeks later. The Micro-CT results showed that the APPL1 overexpression group had more bone trabeculae than the OVX group (Fig. 7a), and the three-dimensional reconstruction of bone trabeculae and bone cortices confirmed that APPL1 overexpression effectively increased not only the number of bone trabeculae in osteoporotic mice but also the thickness of bone cortices (Fig. 7b). Furthermore, the indicators of trabecular bone (BV/TV, BA/BV, trabecular thickness, trabecular number, trabecular spacing and cortical wall thickness) were analysed (Fig. 7c). After the generation of femoral paraffin sections, H&E staining and Masson’s trichrome staining showed a significantly increased amount of trabecular bone in the APPL1 overexpression group, while the number of lipid droplets in bone marrow was significantly decreased (Fig. 7d), consistent with the results of the micro-CT analysis. These findings indicate that increasing APPL1 levels effectively delays the development of osteoporosis in mice and that APPL1 may be an effective target for the diagnosis and treatment of osteoporosis.

## Discussion

**Fig. 8:**
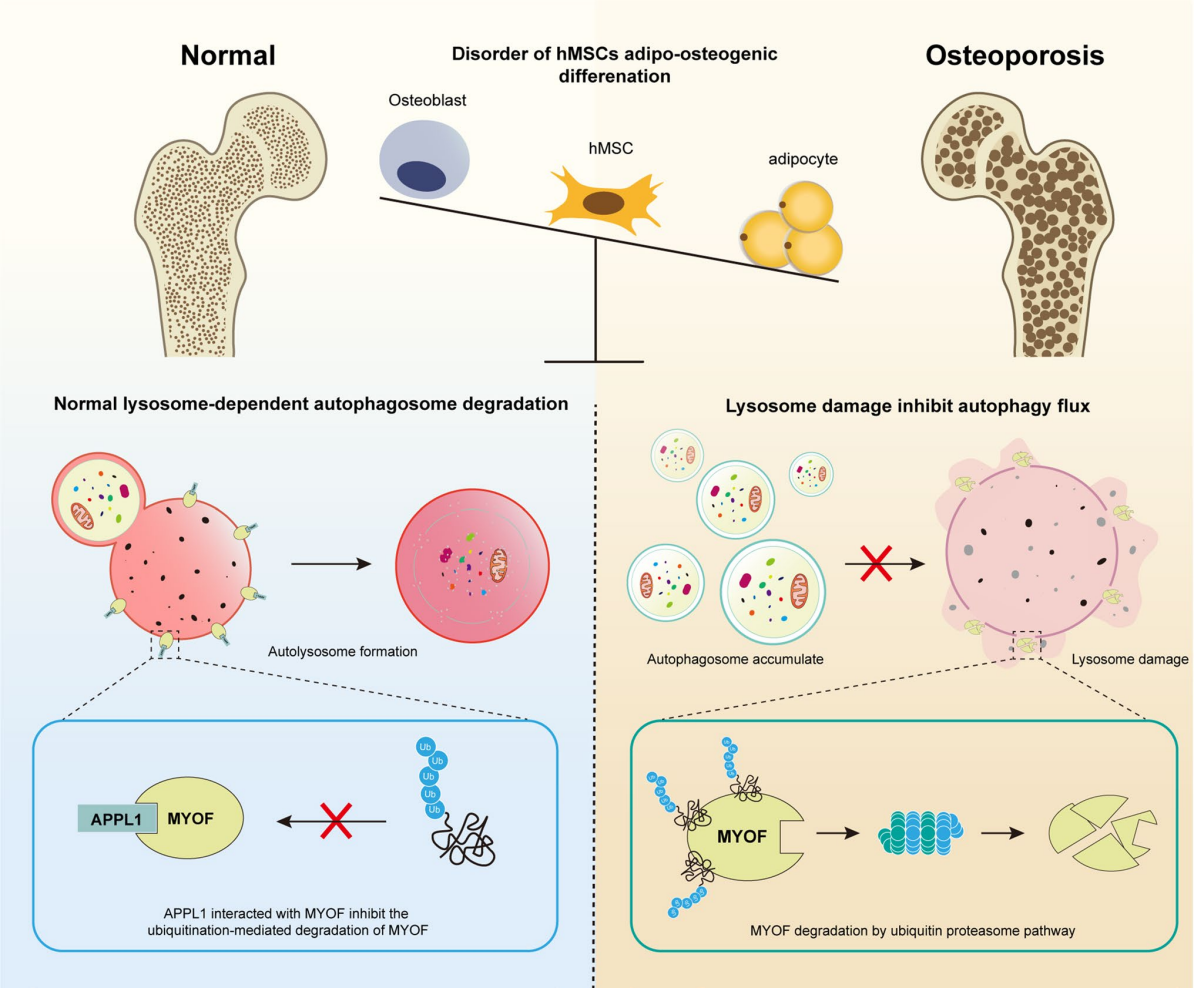
A schematic of the role of the APPL1/MYOF/lysosome/autophagy axis in hMSCs adipogenic differentiation. APPL1 deficiency enhanced the ubiquitination-mediated degradation of MYOF, and downregulation of MYOF expression enhanced the risk of lysosome damage during the adipogenic differentiation of hMSCs. Subsequently, lysosome damage inhibited autophagy flux by suppressing autophagosome degradation, and autophagosomes accumulated inside the cell. Finally, hMSCs differentiation towards the adipocyte lineage was promoted, and the adipogenic-osteogenic differentiation balance was disordered in osteoporosis

In the present study, we revealed that APPL1 expression is downregulated in osteoporosis and confirmed that APPL1 exerts a negative regulatory effect on hMSC adipogenic differentiation which is a protective molecule that maintains the balance of hMSC adipogenic-osteogenic differentiation in osteoporosis. Mechanistically, APPL1 binds to the downstream target protein MYOF and suppresses its ubiquitin-mediated degradation, thus stabilizing lysosome function during hMSC adipogenic differentiation. hMSC adipogenic differentiation was suppressed by maintaining the normal degradation function of the lysosome-autophagy system, and the balance between adipogenic and osteogenic differentiation in hMSCs was maintained to prevent the occurrence of osteoporosis (Fig. 8). Furthermore, an APPL1-overexpressing adenovirus was injected into osteoporotic mice, and the expansion of bone marrow adipose tissue and bone mass loss were diminished that osteoporosis symptoms were relieved, suggesting that strategies targeting APPL1 in hMSCs are effective in osteoporotic therapy. These findings indicate that APPL1 deficiency in hMSCs is an essential cellular molecular mechanism of osteoporosis and that APPL1 plays an important role in suppressing bone marrow adipose tissue growth and maintaining the balance of adipogenic-osteogenic differentiation. Further study of the APPL1 molecular mechanism in hMSCs may contribute to its development as a potential target for the diagnosis and treatment of osteoporosis through hMSC tissue engineering.

Osteoporosis is a prevalent bone metabolic disease. A negative correlation between bone marrow adipose tissue and bone mass has been revealed in long-term research [32]. Ambrosi et al. [33] found that when adipose lineage cells were transplanted into the fracture site of mice, the total BMD at the fracture site was significantly reduced, indicating that adipose tissue restricts bone regeneration. MSCs in bone marrow can differentiate into osteoblasts, adipocytes, and chondroblasts. Adipocytes and osteoblasts are typically derived from MSCs. Generally, osteogenic differentiation and adipogenic differentiation are considered two mutually exclusive differentiation phenotypes, and hMSC lineage differentiation is strictly regulated in time and space to maintain a healthy bone structure. The normal differentiation balance in hMSCs is disturbed by ageing, abnormal hormone regulation or certain stimuli. For example, in individuals with osteoporosis, hMSCs preferentially develop into the adipocyte lineage, resulting in an increase in bone marrow adipose tissue and a loss of bone mass. The molecular mechanism of preferential hMSCs differentiation into the adipose tissue lineage in individuals with osteoporosis remains unclear. Therefore, the mechanism regulating the increased adipogenic differentiation of hMSCs must be elucidated to discover potential treatment targets in individuals with osteoporosis.

Osteoporosis and diabetes mellitus are both age-related diseases. In clinical practice, a relationship between diabetes mellitus and osteoporosis is usually observed, and the proliferation of adipose tissue in bone marrow is also closely related to endocrine regulation in humans [34, 35]. APPL1 deficiency is an important molecular mechanism contributing to diabetes mellitus, and APPL1 plays an important role in alleviating insulin resistance and resisting diabetes [36, 37]. Therefore, we assumed that the occurrence of osteoporosis is also related to APPL1 expression levels. The role of APPL1 in osteoporosis has not been reported in recent studies; therefore, we examined APPL1 expression levels in elderly patients with osteoporosis and a mouse osteoporosis model. APPL1 deficiency in osteoporosis was first revealed in our study, and we propose that APPL1 deficiency is an important molecular mechanism that causes the hMSCs adipogenic-osteogenic differentiation balance to shift towards the adipocyte lineage in individuals with osteoporosis. Although numerous studies have confirmed a correlation between the APN/APNR/APPL1 axis and bone mass, the definite effect of the APN-APNR-APPL1 axis on bone mass remains controversial [38–43]. However, most of the studies were based on interventions targeting APN and ApnR. Due to the effects of different types of molecular crosstalk, we were unable to accurately identify the role of APPL1 in hMSCs. Furthermore, there are many researches revealed the effect of APN/APNR/APPL1 axis in osteogenesis of hMSC. The research of Wu et al. [43] reported that comparing with wild-type (WT) mice, APN-knockout (APN-KO) mice exhibited decreased trabecular structure and mineralization. APN promoted osteogenic differentiation of human adipose-derived stem cells showed in the study of Chen et al. [44]. What’s more, the study of Hu et al. [45] revealed that gAPN increased the capacity of osteoblast differentiation in primary cultured human jaw bone marrow mesenchymal stem cells. After treating with gAPN, the expression level of APPL1 was increased, and the effect of gAPN was reversed by the knock down of APPL1. In conclusion, APPL1 plays a positive role in hMSC osteogenic differentiation. Most previous studies focused on osteoblasts and osteoclasts without further exploring hMSCs adipogenic differentiation. Therefore, we explored the role of APPL1 in the adipogenic differentiation of hMSCs in depth and found that APPL1 exerted a negative regulatory effect on hMSC adipogenic differentiation both in vitro and in vivo. As shown in the study by Lin et al. [39] and Wen et al*.* [46], the adipogenic differentiation capacity of MSCs decreases after APPL1 deletion, in contrast to our study. However, after further analysis, we concluded that the discrepancy might be attributed to the use of hMSCs obtained from human bone marrow in our study, while 3T3-L1 preadipocytes were used in the study by Wen and bone marrow mesenchymal stem cells by Lin. In comparison, research on human-derived cells is more convincing in the study of human diseases as it produces results that are closer to the actual cellular and molecular mechanisms in the human body. In another study of Chen [44] and Hu [45], human adipose-derived stem cells and jaw bone marrow mesenchymal stem cells were used to explore the role of APPL1 on adipogenesis. Their results showed that knockdown of APPL1 promoted adipogenesis, which were consistent with our conclusion. The use of human-derived cells can better reflect the growth state of cells and obtain the experimental data closer to the physiological function in human, which is very suitable for drug testing, cell differentiation and transformation and other experimental research in mechanisms of human disease. Therefore, our research is more convincing to reveal the truly role of APPL1 on adipogenesis in human. Overall, our study might more accurately clarify the role and underlying mechanism of APPL1 in the abnormal balance of hMSC adipogenic-osteogenic differentiation in patients with osteoporosis.

However, the underlying mechanisms by which APPL1 deficiency promotes the adipogenic differentiation of hMSCs remain unclear. Previous research has revealed that APPL1 contains three primary domains: a BAR domain at the N-terminus (N-terminal Bin1/Amphiphysin/RVS 167), a PTB domain at the C-terminus (phosphotyrosine binding) and a PH (pleckstrin homology) domain between the BAR and PTB domains [10]. By performing co-IP and LC–MS/MS experiments, we first confirmed that MYOF is a downstream molecule of APPL1 that plays a crucial role in hMSC adipogenic differentiation. Furthermore, our findings revealed that APPL1 interacts with MYOF via BAR and PH to regulate MYOF protein levels. The ubiquitin–proteasome degradation system and the lysosome-autophagy degradation system are primary protein degradation systems in cells [47]. MYOF has ubiquitination modification sites and is regulated by the ubiquitin–proteasome degradation pathway, according to Qian et al. [48] The regulation of MYOF levels by APPL1 through the ubiquitin–proteasome degradation system in hMSCs was first confirmed in our study.

MYOF was discovered in muscle cells as a member of the Ferlin family and has multiple C2 domains that play a crucial role in cell vesicle transport and plasma membrane repair [16–18]. Gupta et al. [19] showed much higher MYOF expression in pancreatic ductal adenocarcinoma tumour cells than in normal cells. Furthermore, MYOF functions as a membrane repair factor that repairs lysosomal damage and maintains membrane stability, supporting malignant cell survival under high metabolic conditions. However, no relevant reports on the function of MYOF in hMSCs have been published, and researchers have not determined whether MYOF plays a similar role in hMSCs. Therefore, our study focused on the role of MYOF in hMSCs. In our experiment, depletion of MYOF, the downstream molecule of APPL1, diminished lysosome stability and led to lysosomal dysfunction and finally loss, which was observed during the induction of hMSC adipogenic differentiation or lysosomal stress. Based on these results, MYOF plays an essential role in maintaining the normal transport and degradation function of lysosomes during hMSC adipogenic differentiation under lysosome overload conditions. APPL1, the upstream molecule of MYOF, maintains lysosome stability by maintaining MYOF levels; when APPL1 is depleted, hMSCs are more vulnerable to lysosome destruction. Overall, our study is the first to report the molecular mechanism by which APPL1 modulates MYOF levels to maintain lysosome stability in hMSCs.

Lysosomes have long been regarded as housekeeping organelles in cells that maintain homeostasis through synthesis and degradation functions. As a key organelle related to molecular degradation in cells, lysosomes play a critical role in autophagy, and the lysosome-autophagy degradation pathway is an essential intracellular degradation pathway [41]. Although the molecular mechanism by which autophagy modulates hMSC differentiation is unclear, previous research has shown that autophagy plays an important role in maintaining the normal function and differentiation of hMSCs [25]. Ma et al*. *[49] and Liu et al*.* [50] found that the autophagy level of MSCs in elderly mice was significantly lower than that in young mice. Autophagy inhibition not only promotes adipogenic differentiation but also inhibits osteogenic differentiation in MSCs. Qi et al*.* [51] revealed that autophagy was reduced in mice with postmenopausal osteoporosis, and MSC differentiation in mice with postmenopausal osteoporosis shifted towards the adipocyte lineage. Zhang et al. [52] further confirmed that autophagy directly regulates PPARγ, a key molecule involved in adipogenic differentiation. Notably, our research elucidates the distinct molecular mechanism by which autophagy levels are decreased in hMSCs in individuals with osteoporosis. Specifically, APPL1 deficiency decreases the expression of the downstream molecule MYOF in osteoporotic hMSCs to cause lysosomal dysfunction and damage, inhibit autophagy flux, and finally promote hMSC differentiation into adipocytes, resulting in bone marrow adipose tissue hyperplasia and a reduced bone mass. Consistent with the findings from our study, Wu et al. [53] revealed autophagosome accumulation in the absence of APPL1 in mitophagy. Although their research object was macrophages, the results revealed a correlation between APPL1 and the autophagic degradation system. In summary, the results of these studies and our research indicate that a change in the autophagy level is closely related to the occurrence of osteoporosis, and autophagy plays an important role in regulating the balance of the adipogenic and osteogenic differentiation of hMSCs.

Currently, drug therapy is the primary treatment for osteoporosis, and bisphosphonates, denosumab, teriparatide and other major drugs are widely used [54]. Although these drugs are helpful in preventing or relieving osteoporosis, their long-term application is limited by an increased risk of fracture and osteonecrosis, and their benefit is limited to up to 5 years following therapy [55–57]. Therefore, to further investigate whether APPL1 can be a target for osteoporosis treatment, an APPL1-overexpressing adenovirus was injected into a mouse osteoporosis model in our study. APPL1 overexpression in a mouse osteoporosis model successfully inhibited adipose tissue growth and alleviated bone loss. These findings indicate that APPL1 is a crucial target in the progression of osteoporosis, and we hope to treat osteoporosis by targeting this molecule.

In summary, APPL1 plays an important role in maintaining the balance of hMSC adipogenic-osteogenic differentiation in osteoporosis, providing a potential target for osteoporosis diagnosis and treatment. However, several problems remain unresolved in our research. If an hMSC-specific APPL1 conditional knockout mouse is used, the results will be more convincing. The upstream regulatory mechanism by which APPL1 expression is reduced in individuals with osteoporosis remains unclear. Further research is necessary to address these problems.

## Supplementary Information

Below is the link to the electronic supplementary material.

Supplementary file1 (DOCX 23 KB)

Supplementary file2 (XLSX 205 KB)

Supplementary file3 (DOCX 31433 KB)

## Data Availability

Summary data are available from the paper and supplementary materials. The raw data sets generated and analysed during this study are available from the corresponding author.
