# Supplementary material for: Impairment of APPL1/Myoferlin facilitates adipogenic differentiation of mesenchymal stem cells by blocking autophagy flux in osteoporosis
Source: Cell Mol Life Sci. 2022 Aug 19;79(9):488. doi: 10.1007/s00018-022-04511-y (PMC9391247; doi:10.1007/s00018-022-04511-y)
Supplement: Supplementary file 1 — Supplementary file1 (DOCX 23 KB) [file 18_2022_4511_MOESM1_ESM.docx]

| **Supplemental Table S1. Primer sequences for real-time PCR** | | | | |
| --- | --- | --- | --- | --- |
| Primer sequence | | | | |
| APPL1 for human | | Forward |  | 5’-ACTTGGGTACATGCAAGCTCA-3’ |
|  |  | Reverse |  | 5’-TCCCTGCGAACATTCTGAACG-3’ |
| MYOF for human | | Forward |  | 5’-ATCCGCGTCCGAGTGATTG-3’ |
|  |  | Reverse |  | 5’-ACAGACGTGAACTTTGACCAC -3’ |
| PPAR-γ for human | | Forward |  | 5'-GGGATCAGCTCCGTGGATCT-3' |
|  |  | Reverse |  | 5'-TGCACTTTGGTACTCTTGAAGTT-3' |
| C/EBPα for human | | Forward |  | 5'-TATAGGCTGGGCTTCCCCTT-3' |
|  |  | Reverse |  | 5'-AgCTTTCTGGTGTGACTCGG-3' |
| FABP4 for human | | Forward |  | 5’-ACTGGGCCAGGAATTTGACG-3’ |
|  |  | Reverse |  | 5’-CTCGTGGAAGTGACGCCTT-3’ |
| GAPDH for human | | Forward |  | 5'-GGAGCGAGATCCCTCCAAAAT-3' |
|  |  | Reverse |  | 5'-GGCTGTTGTCATACTTCTCATGG-3' |
| APPL1 for mouse | | Forward |  | 5’-AGCCAGTGACCCTTTATATCTGC-3’ |
|  |  | Reverse |  | 5’-AGGTATCCAGCCTTTCGGGTT-3’ |
| GAPDH for mouse | | Forward |  | 5’-AGGTCGGTGTGAACGGATTTG-3’ |
|  |  | Reverse |  | 5’-GGGGTCGTTGATGGCAACA-3’ |

Abbreviations: *APPL1*, Adaptor protein, phosphotyrosine interacting With PH domain And leucine zipper 1 actin beta; *MYOF*, Myoferlin; *PPAR-γ*, peroxisome proliferator-activated receptor gamma; *C/EBP-α*, CCAAT/enhancer binding protein alpha; *FABP4*, fatty acid binding protein 4; GAPDH, glyceraldehyde-3-phosphate dehydrogenase.

| **Supplemental Table S2. siRNA sequences for APPL1** | | | | | |
| --- | --- | --- | --- | --- | --- |
| siRNA sequence | | | | | |
| APPL1-siRNA1 | |  | sense |  | 5′-CCGAAAGGCUGGAUACCUUTT-3′ |
|  |  |  | antisense |  | 5'-AAGGUAUCCAGCCUUUCGGTT-3′ |
| APPL1-siRNA2 | |  | sense |  | 5′-GGAUUUAUGAUGCACAGAATT-3′ |
|  |  |  | antisense |  | 5′-UUCUGUGCAUCAUAAAUCCTT-3′ |
| APPL1-siRNA3 | |  | sense |  | 5′-CCAGCAGUUCAGGAUCCUUTT-3′ |
|  |  |  | antisense |  | 5′-AAGGAUCCUGAACUGCUGGTT-3′ |

| **Supplemental Table S3. siRNA sequences for MYOF** | | | | | |
| --- | --- | --- | --- | --- | --- |
| siRNA sequence | | | | | |
| MYOF-siRNA1 | |  | sense |  | 5′- CAGAUACGUUCAAGUUGUACCTT -3′ |
|  |  |  | antisense |  | 5'- GGUACAACUUGAACGUAUCUGTT -3′ |
| MYOF-siRNA2 | |  | sense |  | 5′- GGCGGAUGCUGUCAAAUAAGCTT -3′ |
|  |  |  | antisense |  | 5′- GCUUAUUUGACAGCAUCCGCCTT -3′ |
| MYOF-siRNA3 | |  | sense |  | 5′- GCGCUGCUAUGUCUAUCAAGCTT -3′ |
|  |  |  | antisense |  | 5′- GCUUGAUAGACAUAGCAGCGCTT -3′ |

| **Supplemental Table S4. siRNA sequences for Si-Ctrl** | | | | | |
| --- | --- | --- | --- | --- | --- |
| siRNA sequence | | | | | |
| Control-siRNA | |  | sense |  | 5′- UUCUCCGAACGUGUCACGUTT -3′ |
|  |  |  | antisense |  | 5′- ACGUGACACGUUCGGAGAATT -3′ |

**Supplementary Table S4****. Characteristics of the study subjects**

|  | Normal control | Osteoporosis Patients |
| --- | --- | --- |
| Number | 6 | 6 |
| Age (years) | 37.64±12.51 | 76±7.49 |
| Sex | Female | Female |
| Hight (cm) | 158.26±6.34 | 157.47±8.09 |
| Weight (kg) | 54.46±6.46 | 58.52±4.63 |
| BMI (kg/m2) | 21.65±0.85 | 23.62±0.56 |
| Age of menarche (years) | 13.41±1.18 | 13.25±1.28 |
| Age of menopause (years) | / | 56.34±3.62 |
| Lumbar spine BMD (g/cm2) | 1.36±0.42 | 0.72±0.33 |
| Lumbar spine T score | 0.49±0.19 | -2.59±0.49 |
| Total hip BMD (g/cm2) | 1.38±0.42 | 0.51±0.24 |
| Total hip T score | 0.47±0.23 | -1.25±0.38 |

Data are shown as the mean ± SD, n=6 in each group. P values for all variables are the result of independent t tests between the control and osteoporosis groups, BMI, body mass index; BMD, bone mineral density.
