# Supplementary material for: Impairment of APPL1/Myoferlin facilitates adipogenic differentiation of mesenchymal stem cells by blocking autophagy flux in osteoporosis
Source: Cell Mol Life Sci. 2022 Aug 19;79(9):488. doi: 10.1007/s00018-022-04511-y (PMC9391247; doi:10.1007/s00018-022-04511-y)
Supplement: Supplementary file 3 — Supplementary file3 (DOCX 31433 KB) [file 18_2022_4511_MOESM3_ESM.docx]

**
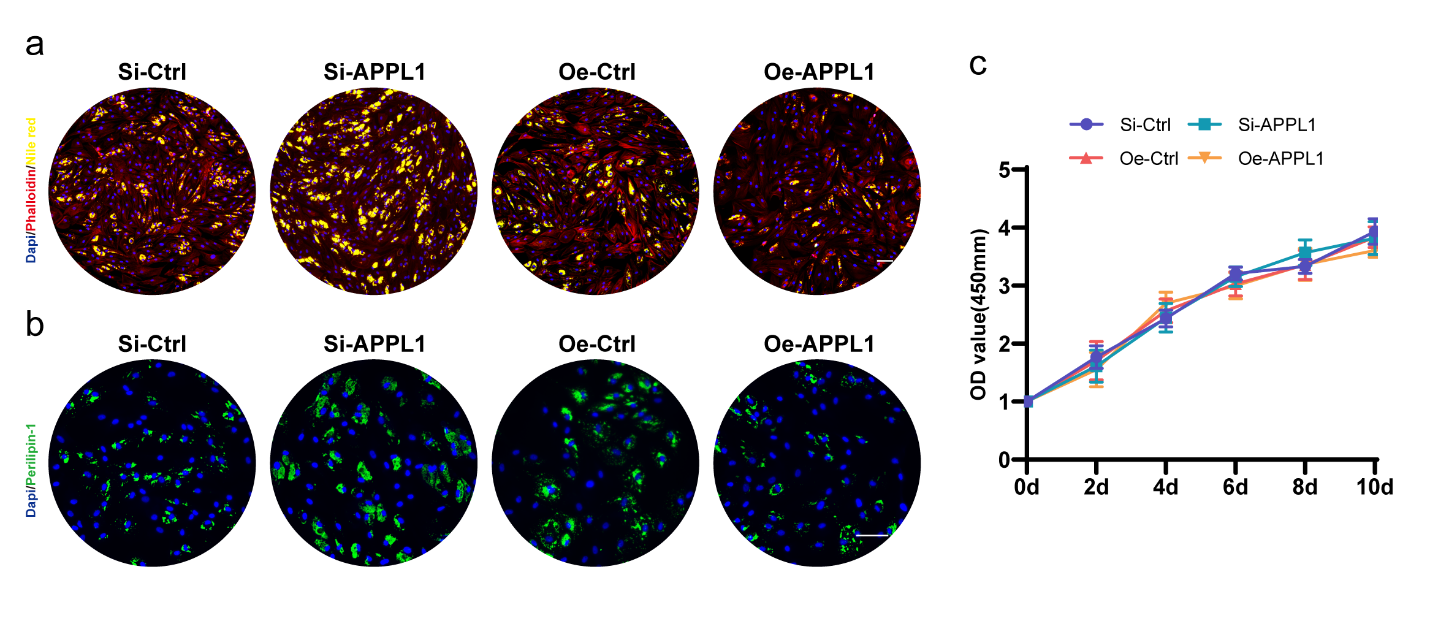
**

**Figure S1**. APPL1 negatively regulated hMSC adipogenic differentiation in vitro. (a) After transfecting SiRNAs and APPL1 overexpression lentiviruses, cells were cultured in adipogenic medium, and Nile red staining (yellow) and quantification on day 10 using fluorescence microscope. (b) Immunofluorescence revealed the level of Perilipin-1 (green) in the APPL1 knockdown group and overexpression group. (c) Cell Counting Kit-8 assays (CCK8) was used to analyzed hMSCs proliferation, and there were no obvious differences. Scale bar = 100 μm. All data are presented as the means ± SD, n = 6 per group. Statistical differences were determined using Student’s t test or ANOVA. ns = not statistically significant, *P < 0.05, **P < 0.01, and *** P < 0.001.


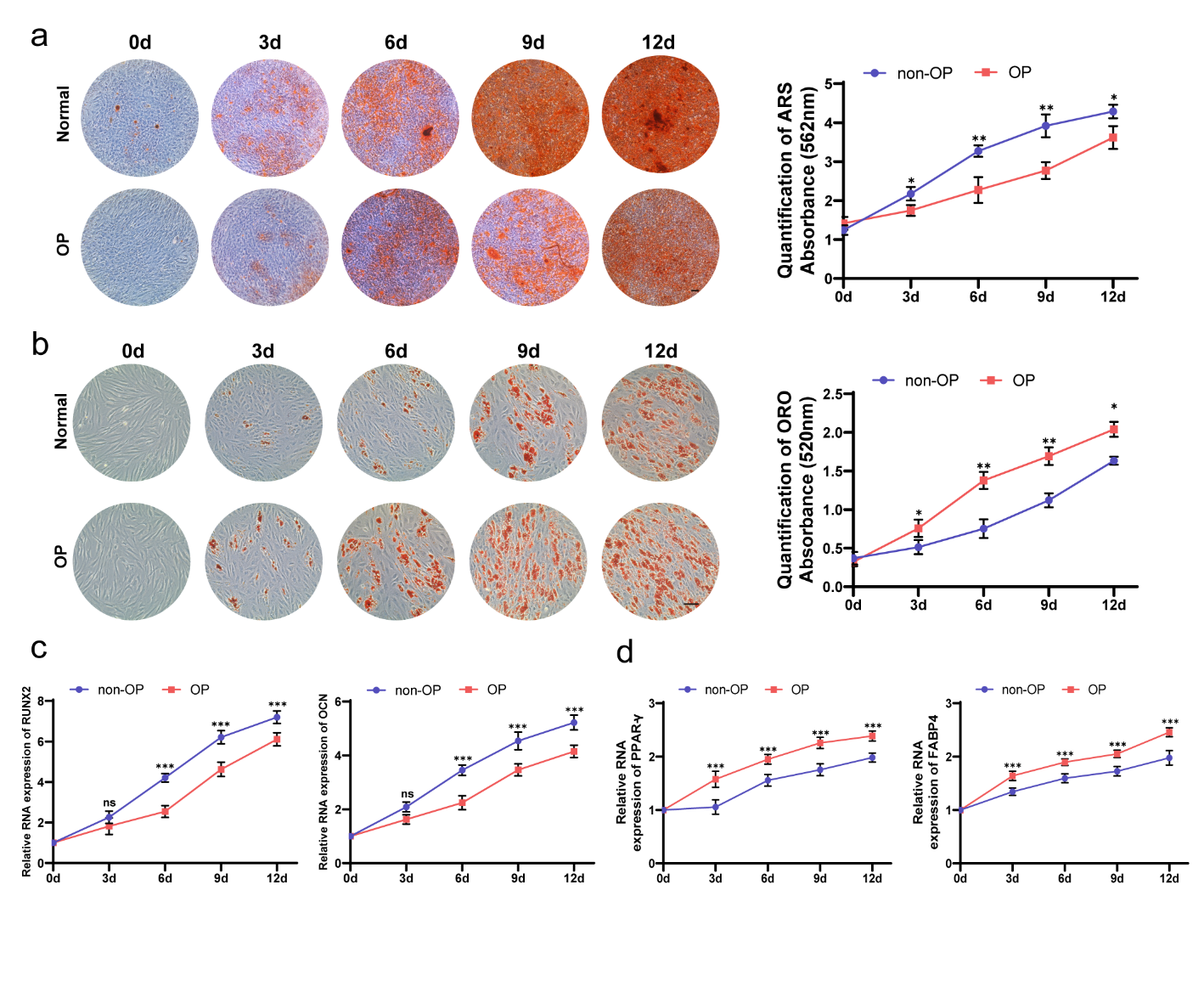


**Figure S2**. hMSC adipogenic-osteogenic differentiation balance disorder in osteoporosis. (a) Alizarin Red S (ARS) staining and quantification showed osteogenic differentiation capability between non-osteoporosis and osteoporosis individuals. (b) ORO staining and quantification showed adipogenic differentiation capability between non-osteoporosis and osteoporosis individuals. (c) Osteogenic markers (RUNX2 and OCN) were detected by qRT–PCR during hMSCs osteogenesis. (d) Adipogenic markers (PPAR-γ and FABP4) were detected by qRT–PCR during hMSCs osteogenesis. Scale bar = 50 μm. All data are presented as the means ± SD, n = 3 per group. Statistical differences were determined using Student’s t test or ANOVA. ns = not statistically significant, *P < 0.05, **P < 0.01, and *** P < 0.001.


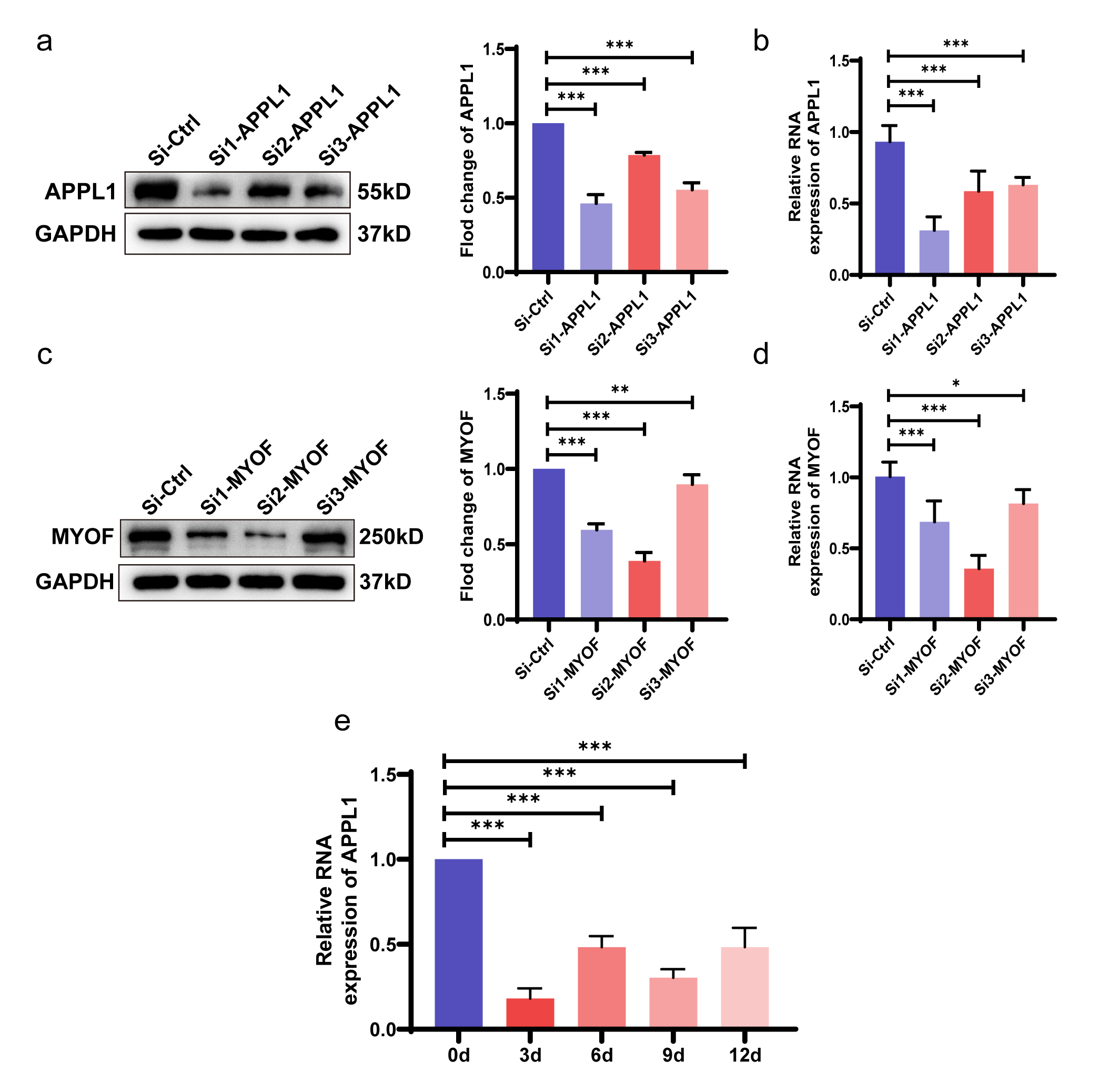


**Figure S3**. SiRNA knockdown efficiency tested. (a) APPL1 siRNA knockdown efficiency detected by Western blot analysis. (b) APPL1 siRNA knockdown efficiency detected by qRT–PCR. (c) MYOF siRNA knockdown efficiency detected by Western blot analysis. (d) MYOF siRNA knockdown efficiency detected by qRT–PCR. (e) the knockdown effect of APPL1 in different timepoints were detected by qRT–PCR. All data are presented as the means ± SD, (a-d) with n = 6 per group and (e) with n = 3 per group. Statistical differences were determined using ANOVA, compared with Si-ctrl group. ns = not statistically significant, *P < 0.05, **P < 0.01, and *** P < 0.001.


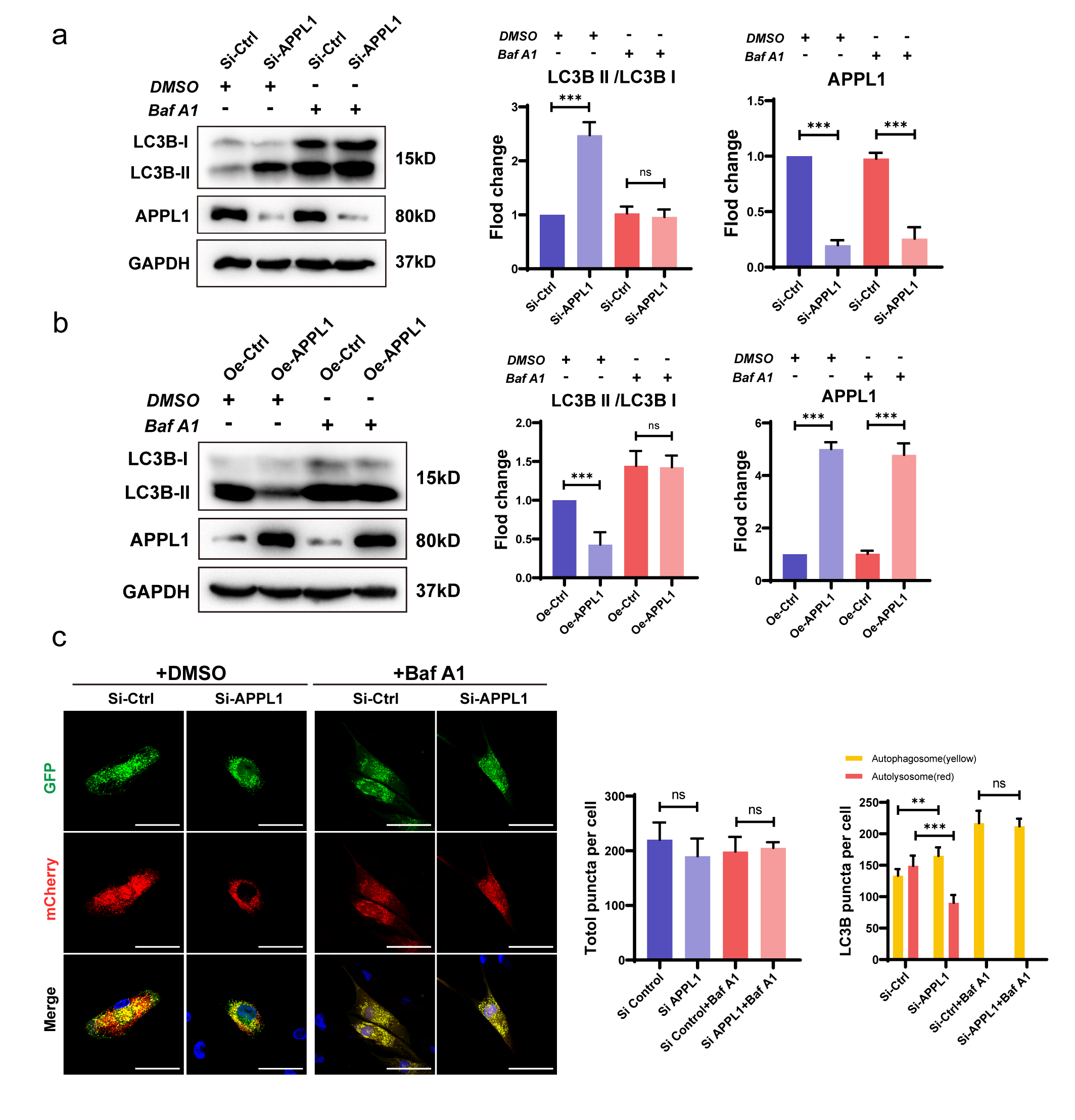


**Figure S4**. APPL1 deficiency inhibits autophagy flux in hMSCs. (a) Western blot analysis of autophagy flux and autophagy activation after APPL1 knockdown under adipogenic induction conditions with or without BafA1. (b) Western blot analysis of autophagy flux and autophagy activation after APPL1 overexpression under adipogenic induction conditions with or without BafA1. (c) hMSCs were infected with a GFP-mCherry-LC3B lentivirus for 24 h, and fluorescent staining was detected using confocal microscopy after APPL1 knockdown. Scale bar = 50 μm. All data are presented as the means ± SD, n = 6 per group. Statistical differences were determined using Student’s t test or ANOVA. ns = not statistically significant, *P < 0.05, **P < 0.01, and *** P < 0.001.
